# The Xenopus FcR family demonstrates continually high diversification of paired receptors in vertebrate evolution

**DOI:** 10.1186/1471-2148-8-148

**Published:** 2008-05-16

**Authors:** Sergey V Guselnikov, Thaminda Ramanayake, Aleksandra Y Erilova, Ludmila V Mechetina, Alexander M Najakshin, Jacques Robert, Alexander V Taranin

**Affiliations:** 1 Institute of Cytology and Genetics, Novosibirsk, Russia; 2 University of Rochester Medical Centre, Rochester, NY, USA

## Abstract

**Background:**

Recent studies have revealed an unexpected diversity of domain architecture among FcR-like receptors that presumably fulfill regulatory functions in the immune system. Different species of mammals, as well as chicken and catfish have been found to possess strikingly different sets of these receptors. To better understand the evolutionary history of paired receptors, we extended the study of FcR-like genes in amphibian representatives *Xenopus tropicalis *and *Xenopus laevis*.

**Results:**

The diploid genome of *X. tropicalis *contains at least 75 genes encoding paired FcR-related receptors designated XFLs. The allotetraploid *X. laevis *displays many similar genes primarily expressed in lymphoid tissues. Up to 35 domain architectures generated by combinatorial joining of six Ig-domain subtypes and two subtypes of the transmembrane regions were found in XFLs. None of these variants are shared by FcR-related proteins from other studied species. Putative activating XFLs associate with the FcRγ subunit, and their transmembrane domains are highly similar to those of activating mammalian KIR-related receptors. This argues in favor of a common origin for the FcR and the KIR families. Phylogenetic analysis shows that the entire repertoires of the *Xenopus *and mammalian FcR-related proteins have emerged after the amphibian-amniotes split.

**Conclusion:**

FcR- and KIR-related receptors evolved through continual species-specific diversification, most likely by extensive domain shuffling and birth-and-death processes. This mode of evolution raises the possibility that the ancestral function of these paired receptors was a direct interaction with pathogens and that many physiological functions found in the mammalian receptors were secondary acquisitions or specializations.

## Background

Immune responses are regulated by a balance of opposing signals delivered from leukocyte surface molecules [1,2]. In the mammalian immune system, several families of activating and inhibitory receptors form an elaborated regulatory network that tightly affects all stages of immune responses. The evolutionary history of this network is poorly understood. While "pairing" of receptors into the inhibitory and activating forms appears to have occurred in invertebrates, there is no clear evolutionary continuity between invertebrate and vertebrate receptor systems [3,4]. Furthermore, ambiguity of relationships is often observed for paired receptors from different lineages of vertebrates [5-8].

Classical Fc receptors (FcR) and killer cell immunoglobulin receptors (KIR) constitute two families that are prototypic for the paradigm of immune regulation through integration of activating and inhibitory signals. Members of each family fall into two main signaling classes. The inhibitory receptors contain ITIMs in their cytoplasmic tails, while the activating receptors associate with the ITAM-bearing transmembrane signal subunits, such as FcRγ (FcRs) or DAP12 (KIRs). FcRs are widely expressed on various leukocyte subsets. They regulate phagocytosis, cytokines release, antibody-dependent cell mediated cytotoxicity, and antibody synthesis [9,10]. KIRs play a crucial role in regulation of human NK cell cytotoxicity via recognition of MHC class I antigens on the surface of target cells [11-13].

During the last decade, it has been recognized that FcRs and KIRs belong to large families comprised of structurally related yet highly diverse proteins. Thus far, eight human and six mouse FcR-like (FCRL) genes have been described [14-23]. Two of them, designated FCRLA and FCRLB according to the new nomenclature [24], are intracellular proteins composed of three Ig-like domains and a C-terminal mucin-like domain. Six human (FCRL1-FCRL6) and three mouse (FCRL1, FCRL5, and FCRL6) genes code for cell surface receptors with the extracellular regions (EC) composed of two to nine Ig-like domains and intracellular regions bearing different patterns of the ITIM-, ITSM- and ITAM-like motifs. Apart from the FcR-characteristic D1, D2 and D3 subtypes, two new structural Ig-like domain subtypes, D4 and D5, have been identified in these proteins. Furthermore, one of the novel mouse genes, FCRLS, encodes a soluble mosaic protein containing a scavenger domain [17,22].

Studies of the KIR family have also revealed its considerable structural and functional heterogeneity. Human KIR-like proteins (KIRL) include cell surface receptors of the LILR (ILT/LIR/MIR) family as well as FcαR, GPVI, Nkp46, OSCAR, Lair1 and Lair2 [25,26]. The LILR family consists of both inhibitory and activating forms: LAIR-1 is an inhibitory receptor, LAIR2 is soluble, the others are activating. Like FcRs but unlike KIRs, the activating LILR receptors, as well as FcαR, OSCAR, NKP46, and GPVI associate with the FcRγ subunit [27-31]. However, the transmembrane regions (TM) of activating KIRLs are structurally different from those of FcRs.

Intriguingly, the repertoires of the FcR- and KIR-related proteins are different from one species to another in higher vertebrates. For instance, each of the six human and four mouse extracellular FCRLs has a unique domain architecture [22,24]. Functional equivalents of KIRs in rodents are C-type lectin receptors of the Ly49 family [32]. The mouse also lacks counterparts of FcαR and Lair-2 and has fewer LILR homologues described as PIRs [25,26]. Profound differences in the KIR and FcR families have been also revealed between mammals and birds. Recent data show that the chicken genome has more than a hundred genes for KIR-like paired receptors known as CHIRs [33-35]. At the same time, a single FcR-related gene has been detected in this species [36,37].

Comparison of the 3-D structure of membrane-proximal domains of FcγRII and KIR2D demonstrated their similar folding and prompted a suggestion that the two families may have had a common origin [33]. This suggestion was made before identification of FCRLs. A later phylogenetic analysis did not provide solid support in favor of homology of five FCRL-characteristic Ig-domain subtypes with the two main domain subtypes of KIR-related receptors [5]. Nevertheless, this idea of the common ancestry of the FcR and KIR families has been revived in modified form after the recent identification of a family of paired receptors called leukocyte immune-type receptors (LITR) in catfish [6]. LITRs are composed of several domain subtypes some of which resemble the FcR-characteristic domains D1D2, whereas others are more similar to KIRL domains. Such composition of Ig domains has been proposed as ancestral for the tetrapod paired receptors [6]. However, weak sequence similarity between LITR and KIRL Ig domains, as well as the absence of the D3, D4 and D5 type domains in LITRs did not allow conclusions about definite relationships of the teleostean proteins with the higher vertebrate FcR and KIR families.

The fact that all known FcR- and KIR-related receptors are primarily expressed in cells of the immune system is consistent with their contribution to the immune regulation. However, the exact functions of all FCRLs and many KIRLs are still unknown. The ambiguity of the structural and functional evolution of FcR- and KIR-related receptors complicates our understanding of how this regulatory network is organized, which factors drives its species-specific changes and ultimately how it may be manipulated for therapeutic purposes.

To gain deeper insight into the evolution of the immunoregulation through paired receptors, we studied the FcR family in the amphibians *Xenopus laevis *and *Xenopus tropicalis*. The data obtained provide evidence in favor of a common origin of the FcR and KIR families and their ceaseless diversification that appears to be caused by very strong natural selection pressure.

## Results

### The FcR family is expanded in amphibians

**Figure 1:**
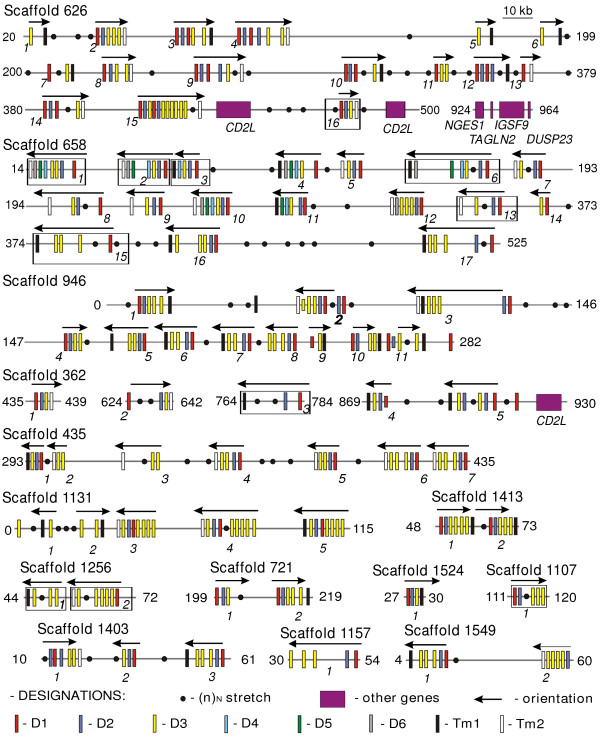
**Genomic organization of the predicted *X. tropicalis *FcR-like genes**. The exons for each particular subtype of the Ig-like domains (D1-D6) are marked by a different color as indicated. Exons for TMs with the NxxR motif (TM1) are in black and those for the TM regions without charged residues (TM2) are in white. The gene models supported by *X. tropicalis *EST cDNAs are boxed. Arrows indicate transcriptional orientation. The genes are designated by their scaffold number and their consecutive position at the corresponding scaffold (version 4.1). Filled circles show position of gaps in the assembly. To conserve space, only fractions of scaffolds are shown; their borders are indicated in kb at the right and left sides.

During our studies of the human and mouse FcR-like genes we observed that EST databases contain numerous *X. tropicalis *and *X. laevis *cDNAs encoding proteins structurally similar to mammalian FcRs and FCRLs. The degree of amino acid sequence identity ranged from 25 to 43% for different domain subtypes. We designated these genes XFL (*Xenopus *FcR-Like). The recent sequencing of the *X. tropicalis *genome provided an opportunity to examine the organization and structure of the XFL genes in more details. We used *in silico *analysis of the version 2, 3 and 4 genomic sequences deposited at the JGI website. We did not consider the consortium gene models in this survey. Direct application of gene prediction programs to genomic sequences often results in erroneous models. To overcome this pitfall, we first identified exons coding for the XFL EC and TM domains using the TBLASTN search with amino acid sequences of the corresponding *X. tropicalis *and *X. laevis *EST cDNAs or mammalian FcR-like proteins. The identified *X. tropicalis *sequences were used in the second round of the computational screening to reveal exons that might have been overlooked in the first round. The procedure was repeated until no novel exons were identified. The exons lacking frame-shift mutations or stop codons were examined for the presence of the AG and GT splice signals matching the phase 1 rule. Thereafter, the gene models were generated using both automatic and manual procedures. The exons for the TM regions served as the gene delimiters. This approach resulted in finding several hundred exons on 33 scaffolds. Of these, 19 scaffolds contained 1–2 exons that may either represent misassembled gene regions, gene fragments or pseudogenes. The exons on 14 other scaffolds could be arranged in at least 75 XFL genes. Fig. 1 shows the predicted organization and exon/intron arrangement of these genes. The exons encoding the signal peptides and cytoplasmic regions are not shown in this scheme because of poor accuracy of their prediction. Nevertheless, in certain cases, such exons could be delineated on the basis of alignment of the genomic sequences with the EST cDNAs. At the time, the EST databases contained *X. tropicalis *cDNAs corresponding to 13 XFL genes. The signal peptides were invariably encoded by two exons, like in the mammalian FcR-like genes. In a fraction of XFL genes, the exons for signal peptides met the rule 30/21 bp (the length of the first/the length of the second exon), which is characteristic of mammalian FCRL1-6, FcγRI and FCRLB genes. The XFL cytoplasmic regions are encoded by one to five exons.

In the mammalian genomes, FcR-like genes are linked to the genes of the CD2 family. Thus, the FCRL6 gene is a part of a conserved syntenic group that includes the SLAMF8 (BLAME), IgSF9, DUSP23, TAGLN2 and NGES1 genes [37]. We found a similar group in the scaffold 626 that contains 16 XFL genes (Fig. 1). The XFL gene 626_16 is located between two CD2-like genes, one of which shows the greatest similarity to mammalian SLAMF8 (BLAME). *Xenopus *homologs of the mammalian IgSF9, DUSP23, TAGLN2 and NGES1 genes are also tightly linked. This conserved synteny taken together with the results of the sequence comparisons (See additional data file 1) and phylogenetic analysis (see below) strongly supports the assignment of the XFL genes as true amphibian homologs of the mammalian FcR genes.

### The XFL receptors are subdivided into two classes

**Figure 2:**
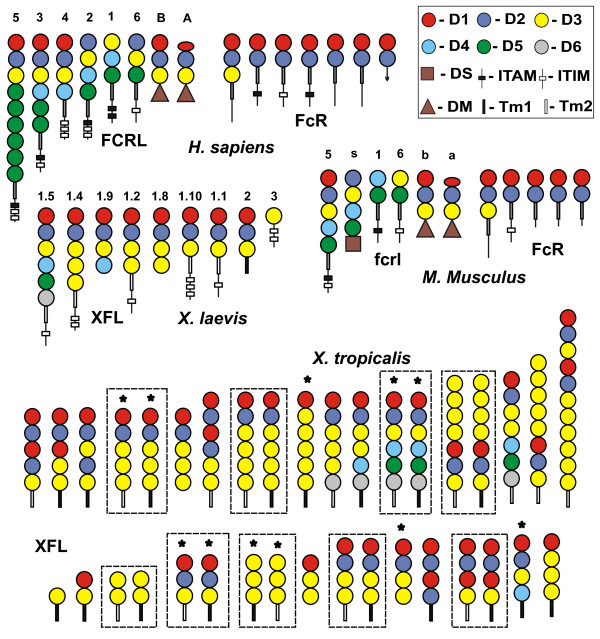
**Schematic representation of domain architecture of human, mouse, *Xenopus laevis *and *X. tropicalis *FcR-like proteins**. The structure of *X. laevis *molecules is deduced from cDNA sequences, whereas the structure of *X. tropicalis *molecules is predicted based on the genomic sequences and confirmed by the EST cDNA sequences (marked with asterisk). The Ig-like domains belonging to the D1-D6 structural subtypes are shown by circles and the TM regions by thick lines. Thin lines and rectangles designate cytoplasmic tails and YxxV/L/I motifs, respectively. The color pattern for the Ig-domains subtypes and transmembrane types are as in Fig. 1. Paired receptors with similar extracellular regions but distinct TM regions are boxed.

**Figure 3:**
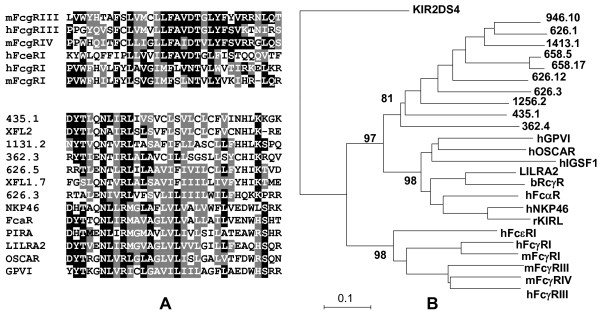
**Alignment (A) and phylogenetic analysis (B) of the deduced TM regions of the *Xenopus *and mammalian FcR- and KIR-like proteins**. All the displayed mammalian members of the KIR family associate with FcRγ subunit. The *X. tropicalis *genes are designated according to the scaffold number and a gene position. Identical and similar residues are shown by white letters on black and gray backgrounds, respectively. The Neighbor-Joining tree of the nucleotide sequences of the TM exons was constructed using the MEGA3 software [39]. The bootstrap values are shown.

With a few exceptions, the predicted XFL genes code for type I cell surface receptors (Fig. 2). Their TM regions fall into two structural types that we designated TM1 and TM2. The characteristic feature of TM1 is the presence of a conserved NxxR motif at the N-termini. TM2 lacks charged residues. Interestingly, TM1 regions are highly homologous to the TM regions of some KIRLs such as LILRA2, PIR-A, NCR1/NKp46, GPVI, OSCAR, and FcαR (Fig. 3). All these proteins are known to associate with the FcRγ signal subunit [27-31]. It is important to stress that TMs of classical activating FcRs are quite different, and bear a typical conserved structural motif (M/L)Fxx(D/N)TxL [38]. Despite the extensive search, we did not find exons for TM regions with such a signature in the *X. tropicalis *genome, or in the available *Xenopus *EST cDNAs. Our phylogenetic analysis supports a close relationship of the TM regions of the XFL and KIRL proteins. As shown in Fig 3b, the NxxR motif-containing TMs and TMs of activating classical FcRs form two distinct clusters. The TM region of DAP12-associating KIR2DS, a member of the human KIR family of MHC class I-specific NK cell receptors, is not related to either of these groups.

Comparison of the *X. tropicalis *genomic and EST sequences showed that the TM1-containing proteins lack a cytoplasmic tail or have a very short one. In every such case, the TM region and the tail are encoded by the same exon. Genes with a TM2 have longer cytoplasmic tails. These tails contain one to three tyrosine-based motifs matching the consensus YxxL/I/V and are encoded by exons separated from TM exons. All these structural features are compatible with the subdivision of the XFL receptors into two functional classes, activating and inhibitory. The number of genes for each class is roughly similar and they are intermingled in the genome (Fig. 1).

### The EC regions of XFLs are highly diverse

A remarkable feature of the predicted XFL proteins is an extraordinary diversity of their domain architectures. Overall 24 different combinations of six structural subtypes of the Ig-like domains in the EC regions of XFLs were found. Five subtypes were assigned to D1-D5 subtypes previously identified in the mammalian members of the FcR family. A sixth subtype appears to be *Xenopus*-specific as no close relatives were found in the protein databases. The D3 subtype domain is most frequent and may be repeated up to 7 times in a protein. Although we cannot rule out that some of the gene predictions result from genome assembly artifacts, we found high similarity between the expressed and genomic sequences. The EST-genome comparisons showed the absence of two exons in the genomic sequences. In both cases, close inspection demonstrated the presence of gaps in the corresponding genomic regions. Eleven of 13 cDNAs fully matched our gene models. This fact, together with the absence of gaps in many predicted genes and reiteration of certain domain architectures two or more times suggest a high degree of confidence in the proposed models. Among the predicted proteins there are typical pairs with identical ectodomains and distinct TM subtypes. (Fig. 1 and 2). The EC regions of 11 XFLs are composed of D1, D2, and D3 domains, like mammalian FcγRI. This is the only EC composition shared by the known mammalian and *Xenopus *FcR-like proteins. If we consider structural subtypes of the TM regions as distinct domains, up to 36 domain architectures may be distinguished among the XFL proteins, none of which are present among mammalian members of the FcR family.

### Lineage-specific expansion of *Xenopus *and mammalian FcR families

**Figure 4:**
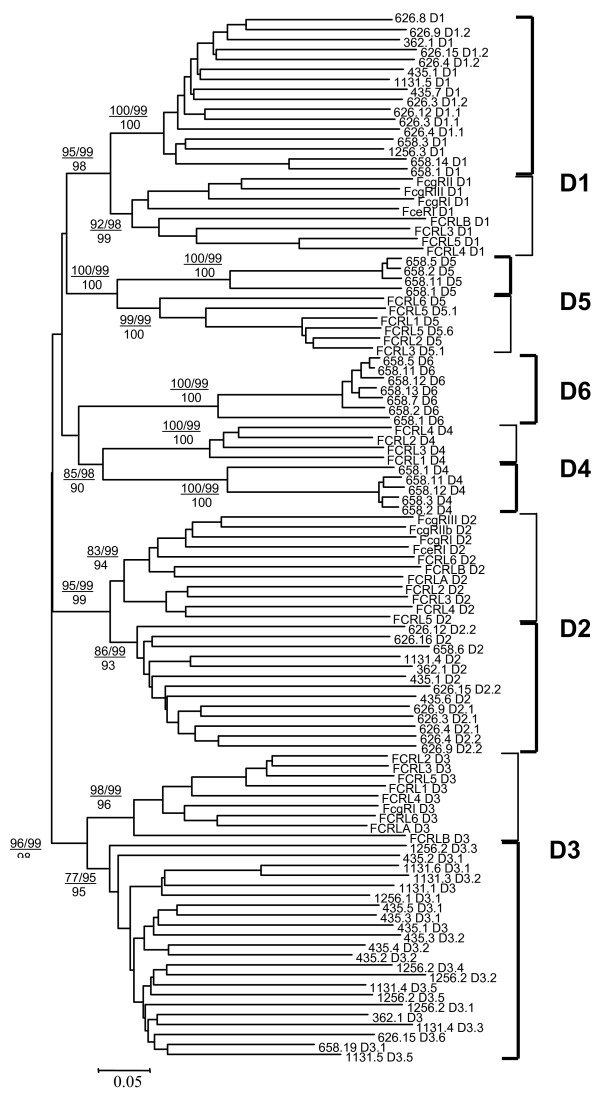
**Neighbor-Joining tree based on the D1-D5 nucleotide sequences of *X. tropicalis *XFLs and human FcR and FCRL genes**. *X. tropicalis *genes are designated according to a scaffold number and their consecutive position (See Fig. 1). For genes containing multiple exons for domains of the same type these exons are numbered according to their position (i. e. D3.1-D3.3). The tree was constructed using MEGA3 software with p-distances for nucleotide sequence sites and pair-wise deletion option. The numbers on the tree represent values for the bootstrap and interior branch tests after 250 replicates.

While the attribution of the XFL proteins to the FcR family is unequivocal according to the reciprocal sequence comparisons and protein database analysis, it remains unclear how *Xenopus *and mammalian proteins are related to each other. To assess such relationships, we generated a series of phylogenetic trees with the MEGA3 software package [39]. For this purpose, amino acid sequences of the Ig domain subtypes from all the predicted XFLs were aligned (see additional data file 1). Trees were generated using the NJ and ME methods. To simplify the trees, the aligned blocks of D1, D2 and D3 domains were reduced by removing redundant sequences with close association. Thereafter, the sequences for all the XFL domain subtypes were aligned together with the domain sequences of the human proteins, and their relationships were analyzed by the same procedure. The final tree is shown on Fig 4. The tree topology supports subdivision of the *Xenopus *and human Ig domains into five common (D1-D5) and one *Xenopus*-specific (D6) subtypes. Most importantly, the tree shows separate clustering of the *Xenopus *and human sequences. This branching pattern suggests that duplications of the FcR-like genes in amphibian and mammalian lineages were lineage-specific and that separation of the mammalian genes into classical FcRs and FCRLs occurred after the split of the amphibian and mammalian lineages.

The relationships among the XFL genes provide evidence for a complex pattern of family evolution in amphibians. Its detailed description is beyond the scope of the present paper and will be published separately. What is relevant to note here, is that the strong association of the amphibian sequences with each other does not necessarily correspond to high level of sequence similarity among them. The cumulative tree illustrates subdivision of the XFL D1, D2 and D3 domains into structural variants whose relationships with each other cannot be resolved. The D1, D2 and D3 domain subtypes fall into 15, 13 and 21 structural groups, respectively. The degree of sequence similarity among the group representatives (32–45% identical residues) is in the range of sequence similarity between XFL and mammalian FcR domains. In the case of D3, the diversity is mainly derived from a small number of genes located in the scaffolds 362, 435, 1131 and 1256. For instance, the extracellular parts of the predicted proteins 1131_3 and 1131_5 are composed of five D3 domains each. These domains are subdivided into four groups. Each of five D3 domains of the XFL 1256_2 represents a distinct structural variant producing a separate branch in the tree. On the other hand, more than 50 proteins encoded by the genes of the other scaffolds have one to seven D3 domains belonging to the same group (group 1). This group may be further subdivided into three main subgroups 1.1, 1.2 and 1.3 and a number of individual members based on the structure of the D2 and D1 domains. For instance, the proteins encoded by the genes on the scaffold 626 have very similar D3 domains but their D1 and D2 domains fall into 8 structural variants with poorly resolved relationships. This fact strongly suggests that intergenic exon recombination was a frequent event in the evolution of the XFL family. Of ten genes containing exons for the D4, D5 or D6 domain subtypes, nine belong to the subgroup 1.1 and one to the subgroup 1.3.

**Figure 5:**
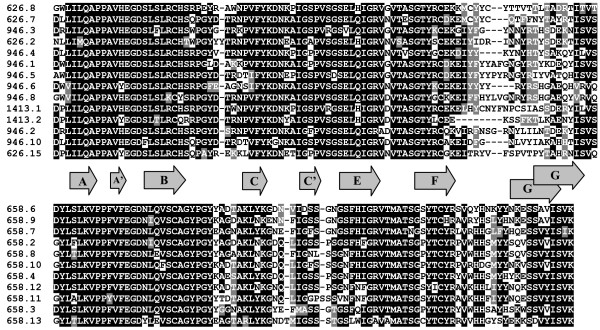
**Alignment of deduced amino acid sequences of *X. tropicalis *D2 domains belonging to subgroups 1.1 and 1.2**. The domains are designated according to the scaffold number (version 4.1) and consecutive position of a gene encoding that particular domain (Fig. 1.). Identical and similar residues are shown by white letters on black and gray backgrounds, respectively. Dashes represent gaps introduced to maximize similarity. Gray arrows indicate predicted β-strands forming Ig-like domain (A-G).

Interestingly subgroups 1.1 and 1.2 differ in patterns of amino acid replacement in the D1 and D2 domains. The D2 domains of subgroup 1.2 are characterized by extensive variation in the length and sequence of the FG loop, the equivalent of CDR3 of the V-type domains (Fig. 5). The region covering the strands C to F is well conserved. In contrast, the D2 domains of subgroup 1.1 show more variation in the region between the C' and F strands. Their F-G region is relatively conserved. The D1 domains of the 1.1 and 1.2 subgroups also display variability at different sites (not shown) suggesting the existence of at least two classes of ligands for the group I receptors. The D1 and D2 domains are implicated in binding to IgG and IgE by classical FcRs. However, the residues known to contact the Fc portion of Ig are not conserved in the XFLs sequences,

### Experimental support of XFL diversity

To gain a deeper insight into structure and expression of the XFL genes, we studied this family in *X. laevis*, a close relative of *X. tropicalis*. In contrast to *X. tropicalis *that has a diploid genome, *X. laevis *is an allotetraploid species. The immune system of *X. laevis *is one of the most thoroughly studied among lower vertebrates [40-42]. Five different *X. laevis *cDNAs for XFL proteins were obtained from the IMAGE consortium and sequenced. More than 30 cDNAs, 11 of which were unique, were additionally cloned from several *X. laevis *cDNA libraries using screening with an exon encoding the D3 domain of group 1 as a probe. Of 16 distinct cDNAs, 9 were full-length, the others were truncated at the 5' end. Nine cDNAs encoded typical cell surface proteins containing TM2-like TM regions and cytoplasmic tails of varying length with one to three tyrosine-based motifs (Fig. 2). The amino acid sequences of two clones had short cytoplasmic tails. Their TM regions contained the NxxR motif and were assigned to the TM1 subtype. One cDNA clone encoded protein lacking TM but containing a typical cytoplasmic tail with two tyrosine-based motifs. Finally, four cDNAs coded for putative secreted proteins composed of the Ig-like domains only. At present, it is unclear whether or not the latter five clones represent alternative transcripts of genes encoding cell surface receptors.

As expected, the initial sequence comparisons demonstrated that most of *X. laevis *derived XFLs may be joined into group 1. Their D3 domains shared 65 to 95% identical residues with each other and with the *X. tropicalis *D3 domains of the group 1 receptors. These proteins were designated XFL1.1 – 1.14. As in the case of *X. tropicalis*, the *X. laevis *group 1 proteins showed variable degree of identity (35 to 85%) in their D1 and D2 domains. Two other proteins were designated XFL2 and XFL3. Their D3 domains shared 35–45% identical residues with each other and with the D3 domains of the group 1 proteins. According to the phylogenetic analysis (not shown), the *X. laevis *XFL2 and XFL3 genes were most similar to the *X. tropicalis *435_1 and 1131_1, 2, 3 genes respectively.

**Figure 6:**
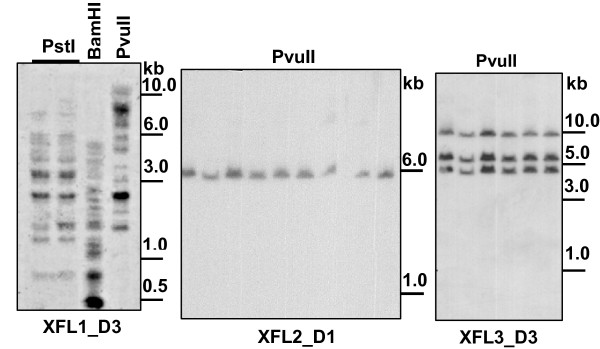
**Southern blot analysis of *Xenopus laevis *genomic DNA**. Hybridizing probes corresponded to the exons for D1 domain of XFL2 or D3 domains of XFL1.1 and XFL3.

To estimate the genomic complexity of the XFL family in *X. laevis*, we performed Southern blot hybridization using the D1-exon of XFL2 and D3-exons of the XFL1.1 and XFL3 genes as probes under non-stringent conditions. Multiple (up to 25) hybridizing bands were revealed on the blots probed with the D3-exon of the XFL1.1 gene (Fig. 6). The XFL2- and XFL3-specific probes revealed one and three hybridizing bands, respectively. These results demonstrated that the cloned XFL genes constitute only a part of the family and that, like in *X. tropicalis*, most of the *X. laevis *XFL genes appear to belong to the group 1.

### *X. laevis *XFL genes are primarily expressed in lymphoid tissues

**Figure 7:**
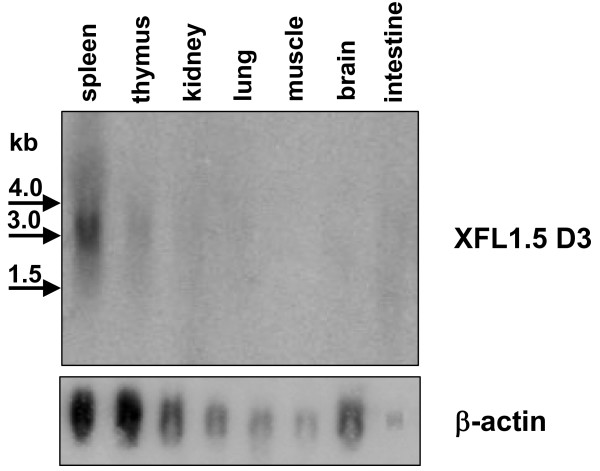
**Northern blot analysis of XFL mRNA distribution in *X. laevis *tissues**. Pooled total RNA from six 6-month old frogs were hybridized under low stringency conditions with the D3 exon of XFL1.5 as an universal probe for group I XFL genes.

To assess the XFL expression pattern, we performed Northern blot hybridization of total RNA from various tissues of adult frogs. The exon for D3 domain of the group I was used as a probe at low stringency conditions. As expected, Northern blotting revealed diffuse bands representing multiple gene transcripts. The highest signal intensity was observed in the spleen and thymus (Fig. 7). To examine expression patterns of the individual genes, we designed gene-specific primers for seven different XFL cDNA. To exclude possible cross matching, the 3'-untranslated sequences were mainly used for the reverse primers. The RT-PCR analysis demonstrated that the tissue distribution of the corresponding mRNA is variable (Table 1). The transcripts of all the genes were mainly detected in lymphoid (spleen, thymus) and non-lymphoid tissues containing cells of haemopoietic origin (e.g., liver, intestine, lung). Expression of the XFL3, XFL1.10 and XFL1.12 genes was detected in brain. The tissue distribution of mRNA in tadpoles was slightly broader. In particular, all XFLs tested except 1.8, were detected in the gills which is known to be a very active immunological site owing to intense blood circulation and high exposure to antigens [43]. Furthermore, expression of the XFL1.8 gene was found only in tadpole spleen. Different pattern of the XFL transcript distribution in adults and larvae suggest that the expression of at least a proportion of the XFL genes is developmentally regulated.

**Table 1:** Variations in XFL expression in *X. laevis *adults (A) and tadpoles (T, stages 46–58).

		XFL						
		
		1.2	1.7	1.8	1.10	1.11	1.12	3
Spleen	A/T	+/+	-/NT	-/+	-/+	+/+	+/+	+/+
Thymus	A/T	+/+	+/+	-/-	+/+	-/-	-/-	+/+
Liver	A/T	-/-	+/NT	-/-	+/+	-/+	-/NT	-/-
Intestine	A/T	-/+	+/+	-/-	-/+	-/-	+/+	+/+
Lung	A/T	+/+	-/-	-/-	+/+	-/+	-/-	+/+
Brain	A	-	-	-	+	-	+	+
Gills	T	+	+	-	+	+	+	+

NT – not tested.

Results are indicative of RT-PCR performed on total RNA extracts from different tissue samples (n = 3).

### TM1 facilitates XFL association with the FcRγ subunit

**Figure 8:**
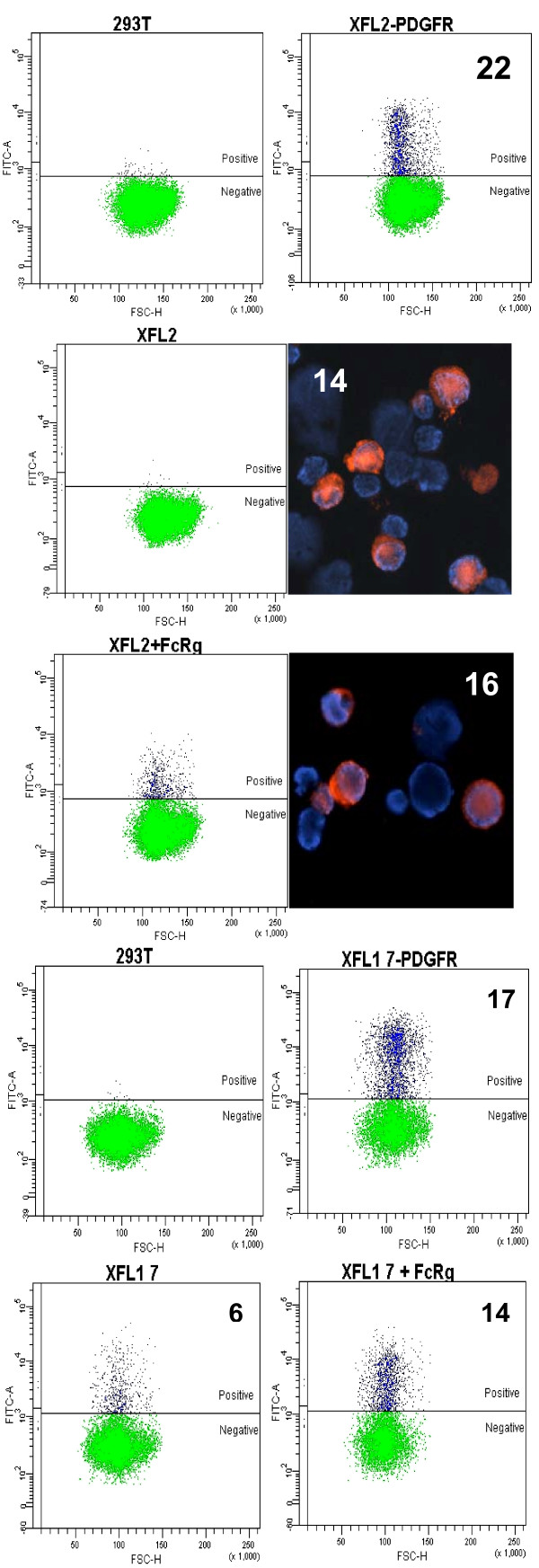
**Requirements for the expression of XFL1.7 and XFL2 on the cell surface**. Epitope-tagged XFL1.7 or XFL2 were ectopically expressed in their native forms, or with the TM regions replaced by that of PDGFR in transiently transfected 293T cells. Effect of co-transfection with FcRγ chain was also studied. Immunocytochemical staining of the XFL2-transfected cells is shown at right. Transfection efficiency is shown as percentage of antigen-positive cells.

The presence of the NxxR motif-bearing TMs in many XFLs suggested that, like the mammalian activating KIRLs, these *Xenopus *receptors may require FcRγ chain for cell surface expression and/or signal transduction. To examine whether this is the case, we generated a series of constructs enabling expression of XFL2, XFL1.7 and the *X. laevis *FcRγ subunit as recombinant epitope-tagged proteins. XFL2 and XFL1.7 were expressed in 293T cells as recombinant hemagglutinin (HA)-tagged proteins containing either their own TM1 regions or the TM region of PDGFR. The *X. laevis *FcRγ chain was tagged with c-myc epitope. Single transfection of XFL2-HA did not induce its surface expression, and the protein accumulated intracellularly as determined by immunofluorescent microscopy of permeabilized cells. However, the surface expression of XFL2 was restored when it was co-transfected with FcRγ (Fig. 8). In contrast to XFL2, XFL1.7-HA was targeted to the surface of the transfected cells in the absence of the adapter molecule, although its surface expression was increased two-fold in the presence of FcRγ. Both XFL2 and XFL1.7 proteins were readily expressed on the cell surface when their EC regions were fused with a TM of PDGFR. These results show that XFL molecules containing a TM1 region with the NxxR motif associate with the FcRγ chain. The surface expression of XFL1.7 is less dependent on the presence of FcRγ chain. This may be explained by divergent structure of their TMs. Such differences also have been observed among the FcRγ-associating KIRLs; FcRγ is critical for surface targeting of LILRA2, but not FcαR or OSCAR [27,29,31].

## Discussion

Comparative studies of mammals and chicken have revealed an unexpected structural and functional variability of the paired receptor families such as FCRLs and KIRLs [22,25,26,34-37]. Studies have also indicated that the repertoires of these families have evolved in a species-specific manner. The evolutionary factors responsible for such diversity remain poorly understood. The recent description of LITRs as putative teleost counterparts of both FCRLs and KIRLs left many questions unanswered, since only a weak similarity of LITRs to the mammalian and avian receptors has been found [6,44]. The present study fills the gap by extending the analysis of paired receptor families to amphibians, the most primitive branch of tetrapods.

In the diploid *X. tropicalis *we have identified at least 75 genes coding for paired FcR-like cell surface receptors. The mere fact of the family expansion is not unusual. Rapid evolutionary change of a gene content known as the "expansion-contraction" or "birth-and-death" process has been documented in many families of immunity-related receptors [45]. What distinguishes the FcR family from many other paired receptor families is the extraordinary structural diversity of its members. Combinatorial joining of six Ig domain subtypes generates as many as 24 EC architectures. When we consider the TM subtypes as distinct domains, the number of the XFL domain architectures increases to 35. None of these variants are shared by either human or mouse homologs, although five Ig-domain subtypes are common for the *Xenopus *and mammalian proteins. Overall, 50 different domain architectures can be defined among human, mouse, and *X. tropicalis *FcR-related proteins.

Except for the D1- and D2-subtype domains no homologous structural elements were found between XFLs and catfish LITRs (not shown). Other Ig-domain subtypes composing EC of LITRs seem to be more similar to the KIRL domains. Such mixed domain composition has been suggested to predate the KIR and FcR families [6]. The XFL data are consistent with the suggestions that the FcR and KIR families share evolutionary roots [33,46]. A strong argument in favor of this model is the fact that activating FcRs use a peculiar TM module (TM1) to associate with FcRγ chain that is homologous to TMs of activating mammalian KIRLs. TM1 appears to be an ancestral element of the primordial FcR/KIR family that has been lost by CHIRs, classical FcRs and KIRs, but retained by XFLs as well as *Xenopus *and mammalian KIRLs.

Although available data do not allow inferring the structure of the KIRL and FCRL ancestor, it is clear that the family evolved by inter- and intragenic recombinations in a species-specific way. The former mechanism gave rapid change in the number of genes per family (birth-and-death), whereas the latter was responsible for extensive domain shuffling. The FcR-related receptors in different vertebrate species are similar in their subdivision into activating and inhibitory forms and predominant expression in lymphoid tissues. However, the ratio of inhibitory to activating members, the cellular distribution, and the exact amount and architecture of ectodomains are unique in each examined species.

What might be the evolutionary forces responsible for this degree of diversity common among the FcR- and KIR-related receptors, and XFLs in particular? We can try to answer this question by inference from the attributed function of the actual mammalian receptors, which is to regulate immune responses. Classical FcRs regulate B cell responses by binding to the IgG and IgE immune complexes, whereas KIRs, or at least their inhibitory forms, regulate NK cell function by binding specifically to MHC class I molecules. However, Ig-binding appears to be a secondary or (derived) specialization, since our previous [37] and present data together with the definition of a chicken IgY receptor as a member of the CHIR family [47] strongly argue that classical FcRs have emerged after the separation of mammals and birds. MHC-recognition as a potential ancient function of FCRLs/KIRLs is more attractive. The ability to interact with classical and non-classical MHC class I molecules is a feature of some KIRLs [11-13,48-50], it has been also suggested for mammalian FCRLs [51] and catfish LITRs [46]. From this point of view the diversification of FCRL/KIRLs may have been driven by the necessity to match the rapid evolution of MHC loci under pathogen pressure of, as it has been suggested for the KIR and Ly49 gene clusters [[32,52] and [53]]. This may be the case for XFLs too as there are at least 20 non-classical MHC class I in *X. laevis *[54].

There are however some inconsistencies with this scenario. First, the scope of variability of domain architectures among FCRLs seems to be excessively high relative to MHC class I molecular structure. Second, the functions of mammalian KIRLs are not limited to MHC antigen-binding. Among ligands of these receptors, there are also collagens (GPVI and LAIR1), IgA (human FcαR), IgG (bovine FcγR2) and integrins (mouse Gp49B1) [55-59]. Human α1-B-glycoprotein, a distant secreted relative of KIRLs, binds to the cysteine-rich secretory protein 3 [60], while its opossum homolog is a snake venom metalloproteinase-neutralizing factor [61]. Due to the independent expansion of KIR-related receptors in mammals, birds [34,35] and amphibians [our unpublished data] it is difficult to determine which of these functions are truly ancient or of ancestral type. Finally, mouse Ly49 is a clear example of the self-MHC recognition served by receptors structurally different from KIRLs and FCRLs.

An alternative explanation for the extraordinary diversity of XFLs and other FcR- and KIR-related receptors may be that they are directly involved in innate immunity. Combinatorial diversity is a hallmark of the immune system and it is usually associated with recognition of pathogens. The capacity of paired receptors to directly bind to pathogens is well documented [62-66]. In the latest of these studies it has been found that mouse PIR-B, and its human relatives LILRB1 and LILRB3 recognize *Staphylococcus aureus *and modulate TLR-mediated inflammatory responses against this bacterium. These facts are usually interpreted in terms of adaptive coevolution of the microorganisms, which implies that the pathogen recognition is a secondary or derived function. However, the extensive variability of the FcR/KIR relatives raises the possibility that these receptors expanded primarily to fight pathogens, whereas the known immuno-regulatory functions may represent secondary acquisitions or specializations.

Depending on the nature of the pathogen and the signal properties of the receptors, it is clear that pathogen-receptor interaction may be either advantageous or detrimental for the host, and as such, may rapidly change the ratio of activating versus inhibitory receptors, as well as their respective amount and specificities. In this regard, a parallel may be drawn with the species-specific expansion of various receptor families in invertebrates that participate in innate immune responses [67,68]. While it may be difficult to obtain direct evidence to support this scenario, it clearly deserves attention. The elucidation of the factors responsible for the diversification of the FcR- and KIR-related receptors may contribute to better understand their function and ultimately develop new therapies based on their immunoregulatory properties.

## Conclusion

Our study shows that in two amphibian species *Xenopus tropicalis and X. laevis*, paired receptors have diversified into a large family of genes, XFLs, preferentially expressed in lymphoid tissues. The extracellular regions of these receptors are composed of one to eleven Ig-like domains belonging to six structural subtypes. A fraction of XFLs use a TM module (TM1) to associate with FcRγ signaling subunit. TM1 is highly similar to TMs of activating FcRγ-associating KIRLs. This fact strongly argues in favor of a common evolutionary origin of the FcR and KIR families. The variation in number and composition of distinct Ig-like and TM domain subtypes generates striking diversity of domain architectures among XFLs. Phylogenetic analysis shows that this diversity emerged in a lineage-specific manner. Classical FcRs and other known mammalian FcR-related proteins appear to be specific to mammals. The continual and extensive diversification of domain architectures in the FcR and KIR families indicates a strong selection pressure not completely consistent with the usual assumption that paired receptors have been primarily selected to regulate immune responses. We propose that FcR/KIR-related receptors might have primarily expanded as pathogen-recognizing components of innate immunity while their known physiological functions have been acquired later in a lineage-specific manner.

## Methods

### Experimental Animals

Adult and larval outbred *Xenopus laevis *were obtained from the *X. laevis *Research Resource for Immunobiology at the University of Rochester Medical Center [69]. Larval development stages were determined according to Nieuwkoop and Faber [70]. All animals were handled under strict laboratory and UCAR regulations. Adults and larvae were euthanized with 0.5% and 0.1% Tricaine methanesulfonate (TMS), respectively.

### cDNA library construction and screening

cDNA libraries from 2 μg spleen total RNA from *Xenopus laevis *adults or froglets (stage 60–62) were constructed using SMART cDNA Library Construction Kit (Clontech). First strand cDNA was amplified using Advantage 2 RCR Enzyme System (Clontech). Size fractionation was achieved by separation on a sepharose columns and 0.5–4 kb cDNAs were ligated into lambdaTriplEx2 arms and then packed with GigapackIII Gold Cloning Kit (Stratagene). Libraries containing 10^6 ^independent recombinant clones were amplified. cDNA libraries from *Xenopus laevis/gilli *LG7 hybrids (from adult spleen or tadpole spleen and liver RNA) were kindly provided by Dr. Louis Du Pasquier (University of Basel, Switzerland). All four cDNA libraries described were screened using^32^P-labeled PCR fragment coding for the first D3 domain of XFL1.2 (279 bp) as described by Sambrook et al. [71]. Plasmids containing cDNA inserts were recovered from isolated positive phages by in vivo excision. cDNAs were sequenced using an automated fluorescent sequencer ABI-Prizm 310 (Applied Biosystems).

### GenBank accessions of cDNA clones

Xenopus EST cDNA clones dc12e01, dai46h06, daa24c04, dab24g06 and NISC_mp06d01 were obtained from the I.M.A.G.E. Consortium [72] through ATCC (USA) or Research Genetics Inc (USA), sequenced as described above and submitted to GenBank. Accession numbers for these cDNAs are [GenBank: AY293300], [GenBank: AY293303], [GenBank: AY293305], [GenBank: AY297106], and [GenBank: EF591296], respectively. [GenBank: AY293301], [GenBank: AY293302], [GenBank: AY293304], [GenBank: AY297104], [GenBank: AY297105], [GenBank: DQ367411], [GenBank: DQ367415] and [GenBank: EF431890–EF431893] accession numbers were assigned to cDNA sequences obtained through cDNA library screening.

### Southern blot analysis

Genomic DNA from Xenopus erythrocytes was isolated as described by Sambrook et al. [71] and digested to completion with restriction endonucleases BamHI, HindIII or PvuII. The digested DNA (10 μg/lane) was separated on 1% agarose gel and transferred onto Zeta-probe nylon membranes (BioRad Laboratories) by the vacuum blotting technique in 0.4 M NaOH. Hybridizations with ^32^P-labeled probes were performed following the membrane manufacturer's recommendations. The probes were PCR amplified fragments coding for the first D3 domain of XFL1.2 (279 bp), D1 domain of XFL2 (215 bp) or D3 domain of XFL3 (239 bp).

### RNA extraction, cDNA synthesis, and RT-PCR amplification

Tissue samples were homogenized in 0.8 mL of Trizol reagent (Invitrogen). Total RNA was extracted according to the manufacturer's protocol. A sample RNA pellet was resuspended in RNase free water and quantified with SmartSpec spectrophotometer (BioRad). 500 ng of quantified total RNA were used to synthesize cDNA with iScript first strand cDNA synthesis kit (BioRad) according to the manufacturer's protocol. Negative RT controls were run for each sample at the same time. cDNA and negative RT control samples were diluted three times to a final volume of 60 μl before proceeding to PCR amplification. For each PCR reaction (30 μl total volume) 3 μl of 2 mM dNTPs, 3 μl of 10× PCR buffer, 10 pmol of each primer, 2 U of Taq DNA polymerase (Life Technologies), and 1 μl of cDNA were used. Then tubes were set for 35 cycles: 45 sec at 95°C, 45 sec at 56–64°C and 30–90 sec at 72°C. The following primers were used for RT-PCR: XFL1.2 forward – 5' GGAAGCTATCAGTGCCAAACA 3', reverse – 5' TGAGTCTCCTGGGAGGACAGA 3', XFL1.7 forward – 5' ACACCAAAGAGGCTGCAGTTC 3', reverse – 5' GATGAGGAGCATCTTCATGGT 3', XFL1.8 forward – 5' ATCGCTATCGCTCTAATGGAGC 3', reverse – 5' CAGTCTCGTGAGATTCAGCCG 3', XFL1.10 – forward 5' GACCAAGTGGACATTGTTGTGC 3', reverse – 5' TTCTCCGGCCTGTCCACCTC 3', XFL1.11 forward – 5' CTCAGGATTCCATCCAAAGTG 3' reverse – 5' CTTGGTCCAGTCCCGCACTG 3', XFL1.12 forward – 5' AGATGCACCCGACAAGTGAAGA 3', reverse – 5' TCAGGACAGCCAGTGCTACTG 3', XFL3 forward – 5' CTACACAAGGATACAACCCTG 3', reverse – 5' TTCTTGGGCATCACCAGAGAG 3', and as internal control, β2M, forward – 5' CCCTTGTGGTGTAACTGTGCTC 3', reverse – 5' GCACACACCAATCAGAAAAAGGAC 3'. Negative (RT) controls were also performed with same primers to control for genomic DNA contamination.

### Northern blot analysis

Total RNA (10 μg/lane) extracted and quantified as described above, was separated on 1% agarose gel with formaldehyde [71] and transferred onto Zeta-probe nylon membranes (BioRad Laboratories) by capillary transfer in 20 × SSC. Hybridization with ^32^P-labeled probe was performed at non-stringent conditions following the membrane manufacturer's recommendations. The probe was PCR amplified fragment coding for D3 domain of XFL1.5 (282 bp). As a control for RNA integrity a probe encoding *X. laevis *β-actin was used.

### Constructions

cDNA regions encoding an extracellular or extracellular plus transmembrane part of XFL2 (or XFL1.7) were cloned using primers with XmaI and PstI (or SalI) sites and ligated into pDisplay (Invitrogen) vector with an N-terminal HA epitope and with or without a C-terminal PDGFR transmembrane domain. The cDNA portions used were 45–809 bp or 45–938 bp for XFL2 cDNA [GenBank: AY293305] and 225–806 bp or 225–938 bp for XFL1.7 cDNA [GenBank: EF591296]. Complete coding region of *X. laevis *FcRγ cDNA [GenBank: EF431895] was cloned using primers with NheI and ApaI sites and ligated into pAP-Tag5 (GenHunter) vector with a C-terminal c-myc epitope.

### Immunochemistry and flow cytometry

Constructions were transiently transfected into 293T cells using Unifectin 56 (IBCH, Moscow, Russia) according to the manufacturer's protocol. Seventy two hours after they were transfected, the cells were used for immunocytochemistry and cytometric analysis. For cell surface staining, transfected cells were washed twice with Wash Buffer (PBS, containing 1% FCS and 0.1% NaN_3_). The cells were first incubated with rabbit anti-HA (Sigma) (anti-hemagglutinin protein) in Wash Buffer for 30 min on ice. Cells were then washed three times with cold Wash Buffer and incubated with goat anti-rabbit Ig-FITC (BD Bioscience) in Wash Buffer for 30 min on ice. The cells were washed three times with Wash Buffer and analyzed using a microscope Axioscop 2 plus and FACSAria cytometer (BD Bioscience). For intracellular staining, transfected cells were smeared on glass slides, fixed with acetone and stained for FcRγ subunit with anti-c-myc monoclonal antibodies (Sigma) and goat anti-mouse IgG-TexasRed (Molecular Probes).

### Bioinformatics tools

Nucleotide and amino acid sequences were analyzed using utilities at the NCBI [73], EMBL [74] and BCM [75] web sites. Amino acid sequences were aligned using Clustal utilities in the MEGA3 software [39] and shaded with the BoxShade program [76]. The nucleotide and amino acid sequences of known genes were retrieved from the GenBank using ENTREZ at the NCBI [73]. The genomic sequences were retrieved from and analyzed at the Ensembl [77,78] or JGI web sites [79]. Homology searches were performed using TBLASTN and TFASTA programs. The GeneScan program [80,81] and the Webgene program package [82,83] were used for the automated gene structure prediction. The XFL-surrounding genes were identified using the Ensembl and JGI utilities and were verified by reciprocal sequence comparisons at the NCBI website using the BLASTP program.

Phylogenetic analysis was performed with MEGA3 [39] for nucleotide sequences of exons and amino acid sequences of domains after alignment with the CLUSTAL option. In certain cases, the CLUSTAL generated alignments were manually corrected. Phylogenetic trees were constructed using the bootstrap and interior branch tests of the Neighbor-joining (NJ) method with p-distances (proportion of differences). Minimum Evolution (ME) trees were essentially the same as the NJ trees in the major branching patterns.

## List of abbreviations

Ig: Immunoglobulin; FcR: classical leukocyte Fc Receptor; FCRL: FcR-Like; KIR: Killer cell Immunoglobulin Receptor; KIRL: KIR-Like; XFL: Xenopus FcR-Like; LITR: Leukocyte Immune-Type Receptors; ITAM: Immunoreceptor Tyrosine-based Activating Motif; ITIM: Immunoreceptor Tyrosine-based Inhibitory Motif; ITSM: Immunoreceptor Tyrosine-based Switch motif; EST: Expressed Sequence Tag; EC: Extracellular region; TM: Transmembrane region.

## Authors' contributions

AVT and JR designed the study, SVG, TR and AMN performed molecular studies, AYE and AVT performed genome search and gene predictions, made sequence alignments and phylogenetic analysis, LVM carried out cell staining and flow cytometry, AVT wrote manuscript, JR helped to draft the manuscript. All authors read and approved the final manuscript.

## Supplementary Material

Additional file 1 **Alignment of deduced amino acid sequences of D1-D5 domains of *X. tropicalis, X. laevis *XFLs and human FcR-related proteins**. *X. tropicalis *genes are designated according to a scaffold number and their consecutive position at the corresponding scaffold (version 4.1). For proteins containing multiple domains of the same type these domains are numbered from N- to C-terminus (i. e. D3.1-D3.3). *X. laevis *domains are designated according to the name of the cloned XFL cDNA (XFL1.1-1.12, XFL2 and XFL3) or GenBank accession number of the EST cDNA (i.e. BU903031). Identical and similar residues are shown by white letters on black and gray backgrounds, respectively. Dashes represent gaps introduced to maximize similarity. Click here for file
