# Supplementary material for: The Xenopus FcR family demonstrates continually high diversification of paired receptors in vertebrate evolution
Source: BMC Evol Biol. 2008 May 16;8:148. doi: 10.1186/1471-2148-8-148 (PMC2413239; doi:10.1186/1471-2148-8-148)
Supplement: Additional file 1 — Alignment of deduced amino acid sequences of D1-D5 domains of X. tropicalis, X. laevis XFLs and human FcR-related proteins. X. tropicalis genes are designated according to a scaffold number and their consecutive position at the corresponding scaffold (version 4.1). For proteins containing multiple domains of the same type these domains are numbered from N- to C-terminus (i. e. D3.1-D3.3). X. laevis domains are designated according to the name of the cloned XFL cDNA (XFL1.1-1.12, XFL2 and XFL3) or GenBank accession number of the EST cDNA (i.e. BU903031). Identical and similar residues are shown by white letters on black and gray backgrounds, respectively. Dashes represent gaps introduced to maximize similarity. [file 1471-2148-8-148-S1.pdf]

D1

```
658.17 D1      GAAARPVLSFSPNWAPILTGDSVTLTCNGAHPAQGNERYTWYRGSNPIPG---VEQQNYTIQGAQREH----SGRYQCQID-TNEKTVALQLPVS
658.16 D1      GAAARPVLSFSPNWAPILTGDSVTLTCNGARPAQGNERYTWYRGSNPIPG---VEQQNYTIQGAQREH----SGRYQCQIG-TSERSAPLQLPVS
658.15 D1      GAAARPVLSFSPNWAPILTGDSVTLTCNGARPAQGNERYTWYRGSNPIPG---VEQQNYTIQGAQREH----SGRYQCQNG-TSERSAPLQLTVS
658.19 D1      GAAARPVLSFSPNWAPILTGDSVTLTCNGARPAQGNERYTWYRDSNPIPG---VEQQNYTIQRAEIEH----RGRYQCQIG-TSERSAALQLTVS
658.3 D1       GAAGRATVSFSPNWASAFTSESVTLTCNVAPSAQGDPPYSWYKDGAPIG----TKQQKYTVVSAQLSD----SGEYQCKSN-SSDRSEAVTLT--
658.9 D1       GAAGRATVSFSPNWASAFTSESVTLTCNVAPSAQGDPPYSWYKDGAPIG----TKQQKYTVVSAQVSD----SGEYQCKSN-SSDRSEAVTLTVS
658.2 D1       GAAGRATVSFSPNWASAFTSESVTLTCNVAPSAQGDPPYSWYKDGAPIG----TKQQSYTIVSAQVSD----SGEYQCKSR-SSDRSEAVTLTVS
658.11 D1      GAAGRATVSFSPNWASAFTYESVTLTCNVASTAQGDPPYSWYKDGAPIG----TKQQKYTIVSAQVSD----SGEYQCKSH-NSDRSEAVTLTVS
1107.1 D1      GAAARPVLSFSPNWAPILTGDNVTLTCNGARPAQGNERYTWYRDSNPIPG---VEQQNYTIQGAQLGQ----SGRYQCQIGERSERSAALQLTVS
658.5 D1       GAAGRATVSFSPNWASAFTSESVTLTCNVASSAQGDPPYSWYKDGVQMK----IRQQNYTIVSAQVSD----SGEYQCKSH-SSDRSEAVTLIIS
658.7 D1       GAAGRATVSFSPNWASAFTSESVTLTCNVAPSAQGDPPYSWYKDGFLIT----IGQQNYTIVSAQVSD----SREYQCKSR-SSDRSEAVTLTVM
658.8 D1       GAAGRATVSFSPNWASAFTSESVTLTCNVAPSAQGDPPYSWYKDGVRMG----IREQNYTIVSAQVSD----SGEYQCKSH-SSDRSEAVTLTVM
658.13 D1      GAAGRATVSFSPNWASAFTSESVTLTCNVAPSAQGDPPYSWYKDGVRMG----IREQNYTIVSAQVSD----SGEYQCKSR-SSDRSEAVTLTVS
658.10 D1      GAAGRATVSFSPNWASAFTSESVTLTCNVAPSAQGDPPYSWYKDGLWIT----IG-QNYTIVRAQLSD----SGEYQCMSR-SSDRSEAVTLTVS
658.12 D1      GAAGRATVSFSPNWASAFTSESVTLTCNVSSSAQGEPPYFWYKDGVRME----TKQQKYTIFNAKKSD----SGVYQCKSH-SSDRSDSVTLTIS
658.6 D1       GAAGRATVSFSPNWASAFTYESVTLTCNVASPAQEDPTYYWCKDGYLIT----IGQQNYTIVSARVSD----SGEYQCKSH-SSGWSEAVTLT--
1413.2 D1      GAAVRPVVSLSPNWTPIFPGESVTLSCNVAPTAQGNLGYSWYWEGQRIR----GDQQSLFIQPARETD----SGDYQCQAG-ASERSAPVTLDIY
946.10 D1      GITVRPGISLSPNWTPIFPGESVTLSCNVAPTAQGNLGYSWYRDEYQIP----GDQQRLVIQPARETD----SGDYQCQAG-ASERSAPVRMEVM
946.11 D1      GAAVRPVVSFSPNWTPIFPGESVTLSCNVAPTAQGNLGYSWYRDGYWIP----GNQQDLDIQAARELD----NGDYQCQAG-ASERSDSVTLEIT
946.4 D1       GAAARPVIFFSPNWNPISEGESVTLSCNVAPTAHGNLGYSWYRYGERIP----GDQQRLVIQAARVTD----SGNYQCQAG-ASERSDSVRLDIR
946.1 D1       GAAVKPVVFFTPNWTPVHQGESVTLSCNVAPTAQGNLGYSWYRDGHQIS----GVQQNLVIQSGTWSD----NGKYQCQLG-TNDISDPVPLHFK
946.6 D1       GAAVRPVVSFSPNWTPIFPGESVTLSCNVAPTAQGNLGYSWYRDGHQIS----GDQQSFVIHSSTSSD----IGNYQCQVG-ANERSNSVRLNFK
946.8 D1       GAAVGAVVSLSPNWTPIFLGESVTLSCNVAPTAQGNLGYSWYRDGHRIS----GDQQSFVIHSSKWSD----IGNYQCQVG-ANERSDPVRLDFK
946.2 D1       -ASFRPVISFYPNWNQVFLGESVTLSCNVAPIAQGNLGYSWYRDGERIP----GDQQRLVIQAARVTD----SGHYQCQAG-ASERSDSVTLDVK
946.5 D1       GAAVRPVVSFSPNWTPIFPGESVTLSCNVAPTAQGNLGYSWYRYGHRIP----GDQQSLTGVMPS--------GYYQCQAG-ASERSDPVRLDIK
946.3 D1       GAAVRPVVSFSPNWTPIFPGESVTLSCNVAPTAQGNLGYSWYRDGYWIH----GDQQSLTEVRHS--------GDYQCQAG-ASERSDSVRLDIK
1413.1 D1      GAAARPVVSFSPNWTPIFPGESVTLSCNVAPTAQGNLGYSWYCNGQRIH----WNQKSLPILPGTEPH----SGDYQCQLA-NYGKSDPMRLDIY
1256.3 D1      GCAVGPHVTIIPPRARALAGENVTLTCHLVPTAQINQSYSWYKDDEKLN----GYEQNYTIHEATRED----SGSYQCQTV-SSNRSEGVTLNVT
362.1 D1       GIAMRPVVSFSPNWATIAKNDSITLTCTVGPTAPLSQRYSWYRDNRRIN----GKERSFIIGKAEEKD----SGNYQCQAG-TSETSDPVRLNVT
362.6 D1       GALVRPVVSFSPNWATIAKNDSITLTCTVGPTAPLSQRYSWYRDNRRIN----GEERSFIIGKAEEKD----SGNYQCQAG-TSERSDPVRLSVT
362.2 D1       GATARPVVSFSPNWAPIAKTDSITLTCTVGPTAPLSQRYSWYRDNERIN----GEERSFIIGKAEEKD----SGNYQCQAG-TSERSDPVRLSVT
362.5 D1       GAFARPVVSFSPNWATISRHETITLTCTVGPTAPLSQRYSWYRDNKRIN----GEERSFIIGKAEEKD----SGNYQCQAG-TSERSDPVRLNVT
362.3 D1       AVAMRPVVSFSPNWATISRHETITLTCTVGPTAPLSQRYSWYRDNETIN----GEERSFIIGKAEEKD----SGNYQCQAG-TSERSDPVRLNVT
435.1 D1       GVTATPWVSFQPDVGKIFFGESITLTCNVASPVQGTPTYSWYRDNKRIS----SGQRLFI-NAALGAN----SGNYQCQAG-TSERSDAVKLKVT
XFL2 D1        -VTARPWVSFQPDVAKIVFGESIALTCNMASPEQGKQTYTWYRDNKRIS----SGQRLYI-NAAFGAN----SGDYQCQTG-TSERSEAVRLEVT
435.4 D1       GSAVRPVVSFLPNWDTIFTGESITLSCDMVSTKHRDQIYYWYKDNISLR----RNQQSLTISSATLED----GGDYQCQTG-TSQRSESARLLIS
435.7 D1       GSAVRPVVLFLPNWDTIFTGESITLRCDMVSTKHRDQIYYWYKDNINLR----RNQQSLTISSATSVD----GGDYQCQTG-TSQRSESARLSIS
435.6 D1       GSAVRPVVSFLPNWDTIFMGDFVSVKCELASTAHKDQIYYWYKDNVYLH----KGQPSFFISSATSED----GGDYQCQTG-TSLKSDSARLSIS
435.5 D1       GSAVRPVVSFLPNWDTIFMGDFVSLKCELSSTAHKDQIYYWYKDNVYLH----KGQPSFFISSATSVD----GGDYQCQTG-TSQKSDSARLSIS
658.14 D1      GSSIKPIVSIDPNWATIFTKESVTLTCNVDPPGPKDQIYYWYRDNQIVPMY--LGTNIVKIDRASKEH----GGSYQCQTV-TSDKSDPVRLQVT
658.1 D1       GDSSIPFVSIDPNWSTVFTKESVTLTCNVDPSGTGEQIYYWYKDNQLIRMY--QKS--FKIDHPKVGY----GGRYQCRTG-TGSKSEAVRLQVS
1131.4 D1      GGAVRPVVSLSPNWANILTGDWVTLTCDVGSTAGTAPRYSWYKDNKMMN--KDTPTKEYIIYYALETN----SGTYQCQTG-TSERSEPVSLSVT
1131.5 D1      GGAVRPVVSLSPNWANILTGDKVTLTCDVGSTAGTAPRYSWYKDNKMMN--KDTPTKEYIIYYALETN----SGTYQCQTD-TSERSEPVSLSVT
1131.6 D1      GGAVRPVVSLSPNWANILTGDWVTLTCDVGSTAGTAPIYSWYKDNEMMNNNKQTNRQSLIINNAKKTD----SGTYQCQMD-TSERSEPVSLSVY
626.10 D1.1    EVSARPVVSLSPNWVPILYGDSVTLTCNLDPNPEGEKRYFWYRDNKKLR----HYKKIYTIYNATVTN----TGNYQCQTD-TSKKSDAVRLAIS
626.12 D1.1    EASARPVVSLSPNWVPILYGDSVTLTCNLDPNPEGEKRYLWYRDNKKLS----HYEKRYTIYNATVTN----TGNYQCRAG-TSKKSDAVRLAIS
626.14 D1.1    EASARPVVSLSPNWVPILYGDSVTLTCNLDPNPEGEKRYFWYRDNKKLN----HYKKSFIIHSASWTQ----KGNYQCRAG-TSEKSDTVRLDIS
626.12 D1.2    EASARPVVSLSPNWTTILYGDSVTLTCNLDPKPEGEKRYLWYRDNEQI-----QYEQSFRIHSAGWKD----KGNYQCRAG-TSEKSDAVRLDTS
626.9 D1.1     GSEIKPVIHLYPNWATVFMYEPVTLACNVTRVVERNLNYSWYKNNRRI-----AQEKTLEIMAAKLKD----SGNYQCRAG-TSDKSDSVRLIVS
626.2 D1       GAAVRPVVSFSPNWTPIFPRESVTLSCNVAPTAQGNLGYSWYRDGHRIP----RDQQNLVIQSRTWSDIQSYRGDYQCQAG-ASERSDPVRLDIK
626.15 D1.1    GAAVRPVVSFSPNWTPIFPGESVTLSCNVAPTAQGNLGYSWYRDGERIR----RDEQ----NLTGVTD----SGDYQCQAG-ASERSDPVTLDVS
626.8 D1       GAAVRPVISLSPNWTPIFPGESVTLSCNVAPTAQGNLGYSWYRDGYWIR----GDEQ----SLTGVTD----RGDYQCQAG-ASERSDNFRLDVS
626.3 D1.1     GAAVRPVISLSPNWATILQGESVNLTCNVDAAVQQNQ-YCWYKNGEQI-----HSGRTITIQDARESN----SGNYQCRAG-DSDMSDIVRLDVK
626.3 D1.2     ---VRPLLSLSPNWVPLLTEDFILLTCDVASTHGWKQKFYWYKDGRSLG----WNQGSLIVS-ADEED----SGNYQCQTD-ASDRSDPLRLDVR
626.4 D1.1     EEVVRPLVSFSPNWAVVLPGDSVTLSCNLDPVEPANHTYTWYKNGAIIN----KHQQSFEIQSASWVD----RGHYQCQTN-YSAKSDSLELEVS
626.9 D1.2     EERLKPLISLSPQWGTILTDDFVILTCNAASAARGTRTYSWYKNGNWIS----GNQQSLEIENAEVTD----RGDYQCQAG-ASQRSAPVTLDVR
626.10 D1.2    GAGTKPEVAINPESDPILVMDSVTLSCNVAPTLPKSRRYIWYRDGDWIP----KERKTFILYRVQKKD----IGHYQCQIP-GSERSDPVRLHVK
626.11 D1      GGGTKPEVAINPQRGPILETDSVTLSCNVAPTTSESRRYIWYRDGGWIP----KVQKTFTLHRVQEKD----SGHYQCQIP-GSERSDTVRLNVK
626.13 D1      GGGTKPVVSINPEWDPILETDSVMLSCNVAPSTPERQRYIWYRDGGWIP----KEQKTFTLDRAQEKN----SGKYQCQIP-GSERSDPVRIDIK
626.14 D1.2    GAGTKPEVAINPQRGPILVMESVTLTCIVASTTSESRRYIWYRDGDWIP----GDLKFFTLLRAQEKD----SGKYQCQIP-GSERSDPVRIDVE
626.15 D1.2    GAAARPVLSVSPNWAPILTGDSVNLTCNGARPAQGNESYTWYRGNSQRTG---VERQTYVIHRARREQ----SGRYQCQID-AGDRSAAFQLTVS
626.16 D1      GASGKPVVSFSPNWATILTYESITLTCDVGSPAQRDQRFTWYRNHRSLN----INEQSYTVKYAEQEH----IGDYQCRAG-TGDISEAVRLEVS
626.4 D1.2     DSTIRPVIQFSPKWATILLGDIVTVICSVALTMPSKQRYSWYRDDEWIN----EMGQNLVILKADMHH----GGNYQCQVN-AGGRSDPVQLDVS
XFL1.8 D1      -TSGTSVISLSPNWNPIFIGDSVTLTCNVAPTAQGNLEYSWYRDGHRIS----GDQQRLVIHHGIWSD----KGQYQCQAG-ANERSDPVHLDLK
XFL1.9 D1      GDSSIPFVSFDPNWATVFTKESVTLTCNVDPPEPRDQIYYWYKDNNFIRMY----QKSFKIDNPIVKY----GGMYQCQIS-KGNKSEAVRLQVS
XFL1.10 D1     GAAVKPVVSFNPNWGTVLAYESVTLTCNVAPPAQREQKYHWYRNNRNLT----INDQSFTVNYAEEKD----IGDYQCRAG-NGDISEAVRLEVS
XFL1.4 D1      AGAAKAIVIFSPNWATVFTKESVTLTCHVDSSAG---GFSWYKDGVQIR----S-EQKYTIGSAQVSD----SGRYQCKSQ-SSDRSDSVTLTVS
XFL1.6 D1      GAAVRTIVNFSPNWATIFTYESVTMTCNVDSSAQGDLGYSWYKDGSLIS----SGQQKYTIDTAQESH----SGEYKCKTR-NSDLSEAAKLTVS
CF270577 D1.2  GALVRPVVSFSPNWATISLHDSITLTCNVSSTSLLNQRYSWYKDNERIK----GEEQNFRIENAEEKD----SGNYQCQTG-TSERSDPVRLDVT
CF285478 D1    GAAVRAVISFSPNWTPIFTGESVTLTCNVVPTAQGNLEYSWYRNGHRIP----GDQQRLVIGSGIQSY----NGNYQCQTG-VNERSDPVRLDFK
CF271510 D1    GAAVRPIVNFSPNWATIFTSESVTMTCNVDSTAQGDLGYSWYKDGSLIG----SGQQKYTIDTAQESH----SGEYKCKTR-DSDLSEAGKLTVS
CF270196 D1    GAA-KAIVIFSPNWATVFTKESVTLTCHVDSSAG---GFSWYKDERWIS----S-EQKYTIGSAQVTD----SGRYQCKSQ-SSDRSDSVTLTVS
EG578391 D1    GAGTKPVVSIKRQWNTALLMNFVTLTCIVTSTVPERWRYVWYKDGDWIP----EEQNTFTLQHAQEKD----SGNYQCQTP-GSERSDPVRLDVE
FcgRII D1      -APPKAVLKLEPQWINVLQEDSVTLTCRGT-HSPESDSIQWFHNGNLIP----THTQPSYRFKANNND----SGEYTCQTG-QTSLSDPVHLTVL
FcgRIII D1     EDLPKAVVFLEPQWYSVLEKDSVTLKCQGA-YSPEDNSTQWFHNESLIS----SQASSYFIDAATVND----SGEYRCQTN-LSTLSDPVQLEVH
FCRLB D1       ATLEKPILSLHPPWTTIFKGERVTLKCDG—YHPLLLELQPISTLWYLGH-----LLLPSHKKSIEVQT----PGVYRCQTR-GAPVSDPIHLSVS
FcgRI D1       -DTTKAVISLQPPWVSVFQEETVTLHCEVL-HLPGSSSTQWFLNGTATQ----TSTPSYRITSASVND----SGEYRCQRG-LSGRSDPIQLEIH
FceRI D1       -VPQKPKVSLNPPWNRIFKGENVTLTCNGNNFFEV-SSTKWFHNGSLSE----ETNSSLNIVNAKFED----SGEYKCQHQ-QVNESEPVYLEVF
FCRL3 D1       GVAPKAVLLLNPPWSTAFKGEKVALICSSISHSLAQGDTYWYHDEKLLK------IKHDKIQITE-------PGNYQCKTR-GSSLSDAVHVEFS
FCRL5 D1       ARTPRPIIFLQPPWTTVFQGERVTLTCKGFRFYS-PQKTKWYHRYLGKE-----ILRETPDNILEVQE----SGEYRCQAQ-GSPLSSPVHLDFS
FCRL4 D1       AAAHKPVISVHPPWTTFFKGERVTLTCNGFQFYA-TEKTTWYHRHYWGE-----KLTLTPGNTLEVRE----SGLYRCQAR-GSPRSNPVRLLFS
```

D2

```
658.19 D2      --VILQAPP-GAHEGEELTLRCYTRPEYKTTNTTVFYKNKL----TIRPLGSDS-LVLGGAQTHMSGPYSCQTKLYIKSVGVTN---KYSSPEEHLSVT
1107.1 D2      --VILQAPP-GAHEGEELTLRCYTRPEHKTTNTTVFYRNNS----IIRPLGSDS-LVLGGAQTNMSGPYSCQTKLHDKSQGVSN---PYTSAEEHLSVT
435.4 D2       EYVILQKPP-VVYEGETLFLRCHSYPGFQS-KETTFNKNSK----LLGSVG---DTYSGDS---VTGTYTCSKYVHHNLRYYS------HTAEAHITVT
435.7 D2       EYVILQKPP-VVYEAETLFLRCHSYPGYHS-KETTFYKNSK----LLGSVG---DTYRGDS---VTGTYTCSKYVYHGLHYYS------HTAEAHITVT
435.5 D2       EYIILQKPP-VVYEGDTLFLRCHSFPGYKS-KETTFYKHGT----VLRSLG---DLYSGDC---TTGTYTCSKHVHHTLRFHF------YTAEAYISAK
435.6 D2       EYIILQKPP-VVYEGDTLILRCHSIPGYKS-KQTTFYKYGT----VLRSLG---DLYSGDC---TTGRYKCSKYVHHAQGFHV------FTAEAYISAK
626.15 D2.2    EKLILQAPP-GVHEGDELILRCHHPPDYRTSDTTFEFREEP----INRSLGSDSVLPLGKAQANMSGPYRCKTKLYDKFHDITH---NYTSPVEHLSVT
946.1 D2       DWLILQAPP-AVHEGDSLSLRCHSRPGLDA-KKPVFYKDNK----AIGPPVSGSELQIGRVGVTASGTYRCDKEIYFYYAFGN--GYRTYKDEQYISVS
946.4 D2       DLLILQAPP-AVHEGDSLSLRCHSRPGYVT-RNPVFYKDNK----PIGPPVSGSELQIGRVNVTVSGTYGCEKDIYYKYN------YHTYSAKQYILVS
946.10 D2      DLLILQAPP-AVHEGDFLSLRCHSQPGYDT-RDTVFYKGNK----AIGSPVSGSELQIGRVDVTASGTYRCAKEIDFGNL------VYIAKAHHTISVS
626.7 D2       DLLILQAPP-AVHEGDSLSLRCHSQPGYDT-RNPVFYKDNK----AIGSPVSGSELQIGRVNVTESGTYRCDKEMCYYCQT-----FFNYTAYRTISVS
626.8 D2       GWLILQAPP-AVHEGDSLSLRCHSRPEYRA-WNPVFYKDNK----PIGSPVSGSELHIGRVGVTASGTYRCEKKMCYYCYTT----VTTLTADRTITVT
1413.2 D2      DPLILQAPP-AVYEGDSLTLRCQRRPGYDT-RNPVFYKDNY----AIGSPVSGSELHIGRVDVTASGTYLCEEKSSFKTLK----------AENYISVS
946.2 D2       DRLILQAPP-AVHEGDSLSLRCHSRPGYDS-RNPVFYKDNK----AIGFPVSGSELQIGRADVTASGTYRCQKVIRFNSGRN---YLILNTDEKYISVS
946.6 D2       DWVILQAPP-AVYEGDSLSLRCHSRPGFEA-GNSIFYKDNK----AIGSPVSGSELQIGRVNVTASGTYKCEKEIYYYY------RYRSHGAEQHVRVQ
946.8 D2       GWVILQAPP-AVHEGDSLSLKCYSRPGYDT-RNPVFYKDNK----AIGSPVSGSELQIGRVDVTASGTYGCKKEIFFHYLVGN--RYRSHGAEQYVRVQ
946.5 D2       AWLILQAPP-AVHEGDSLSLRCHSRPGYDT-RDTIFYKDNE----PIGSPVSDSELQIGRVGVTASGTYRCDKEIYFYYPYRN--GYRIYVATQHISVS
626.2 D2       NLLIMQAPP-AVHEGDSLSLRCHTWPGYYYTRNPVFYKDNE----AIGAPVSGSELHIGRVNVSASGTYSCEKEIYIDR--IN--NYRTYSDEKTISVS
626.15 D2.1    DPLILQAPP-AVYEGDSLSLRCHSQPAYRE-KKLVFYKDNE----TIGPPVSGSELQIGRVNVTASGTYRCGKEITRYVFSP----VTPYTAHRNISVQ
626.4 D2.2     DWLILQAIS-IVHEGDSLFLRCHSRPGYDA-KNTVFYKDNE----AIGSPASESVLHIGRVDVTAAGTYYCEKEVFYYAY------YRKFNAKGNISVL
626.3 D2.2     EHLILQTPP-FIHEGDFLLLRCYSSSEYNE-TNITFYKDGN----IIQSPITDSVSNLGRVSLSASGKYRCEKQIYYNELHYN------LSAEVNVSVS
1131.5 D2      GPLILQAPP-YVYEGDSLTMRCYSESGWD----TMFHKDNN----PVAPPYGD-QLHIEKANMTVTGTYRCSKRNIYTYIQYSYKPYDLQTVEVFISVR
1131.6 D2      GPLILQAPP-YVYEGDSLTMRCYSESGGD----TVFYKDNN----TIKPMNGD-FLHMNQVNVTVTGTYRCSKDKTY-----------QTDDVFISVR
1131.4 D2      GPLILQAPP-YVYEGDSLTMRCYSKSGGD----TVFYKDNN----PVAPTYGD-QLHTEGVNMTVTGTYRCSKGKTY-----------QSDSVFISVR
626.4 D2.1     DTVILQVPP-RINEGDPLALRCHDRPGFDI-QSTLFYKDNE----AIEIPVTDSVLHLGKANRNMTGIYKCKGFYHGKMYWS---------SKRFISVT
626.9 D2.1     GKVILQVPP-RINKGDTLLLRCHSRPKDNA-RNALFYKGNM----SVQHLGADSVLRLEEVDGDATGSYRCSIMIGLQIYLS---------NEEFISVT
626.9 D2.2     DFVILQAPP-SVYEGDSLTLNCYSLPGYNG-RNTVFYRDNS----TLETQPTDSYLRIGTVNWNTSGTYRCAKEVNYYDYFYHSYGFYTHSAEVIISVS
626.10 D2      GPVILQAPP-SVREGDSFSLRCYSGQHNST-ENAVFTKNGV----AMESTGAILDLQPDTN---TTGTYKCSKPLSMFPDSGH-------SGEEFINFE
626.12 D2.1    GPVILQAPP-SVHEGDSFSLRCYSGRHNST-ENAVFTKNGV----AMESTGAILDLQADTN---TTGTYNCSKPLSMFPYSGH-------SGEEFINVE
626.12 D2.2    GPVILQAPP-SVHEGYSFSLRCYSRRHNST-ENAVFTKNGV----AMESTGAILDLQADTN---TTGTYQCSKPLSMFPYSGH-------SGEEFINFE
626.3 D2.1     DDIILQAPP-RINIGDFLTLRCHSRPTKGV-ITTIFFHKDK----IVQSPVTGSVLHVGNVGRNESGTYRCAKKFDRDVLLF---------AEEFISVR
362.1 D2       DRLILQAPP-SVYEGDPLTLRCYSP--YTKPVNATFYKDNE----TISS-LNDS-LTLGASVG-LAGTYRCKVCQPTEFNHSS----PYSSAGIAITFQ
362.5 D2.1     DRVILQAPP-SVYEGDPLTLRCYSP--YTKAVHVTFYKDNV----TISSSPTDS-LTLGASVG-MAGTYRCKAFKLTEFNYSS----PYTSADTAITIQ
362.2 D2       DPVILQAPP-SVYEGDPLTLRCYSA--YIKPVNVTFYKDNV----TISSSPTDS-LTLGASVG-MAGTYRCKAFQPTEFNHYA----PYSSPDIAITIQ
362.5 D2.2     DRVILQAPP-SVYEGDPLTLRCYSP--YTKAKEVMFYKDNV----MISSSPTDS-VTLGASVG-MAGTYRCRAILLLELNHYA----PHSSVDIAITIQ
362.3 D2       DRVILQAPP-SVYEGDPLTLRCYNP--YTKAQNVTFYKDNV----TISSSLTDT-LTLVGSMA-MNGTYTCIKYLQRHAIRYS----PYTSAEIVITIQ
362.4 D2       ERVILQAPP-SVYEGDPLTLRCYTP--YYTAQNVTFLRDNV----IISSSLTDT-LTLVGSMA-MNGTYKCKKNLQHHTNHYS----PYTSAETVITIQ
CF270577 D2    ERLILQAPP-SVYEGDPLTLRCYTP--NSIGKEVTFYKDNV----IISSSPTDT-VTLDTSVG-MTGTYRCKALLPSELHCYS----HYTSAEIVITIQ
626.14 D2      GPVILQAPP-SVREGDSLYLRCHAQFGYYI-RNARFYKNGV----LINSFGAGSAYRLEKASLDTTGTYKCAASADTSPAVGN-------SKEEFIYVG
626.16 D2      AHVILQAPP-MVFEGDTLSLRCHSYDKYPA-SETKFYKDHK----EIKSLGTASEFLMGKVNAEMSGLYKCSRRIMYHSSLHN------YSDQVDISMQ
658.6 D2       DYLSLKVPP-FVFEGDNLQVSCAGYPGYYA-DTAKLYKGDN----VID-SSGNGSFHIGRVTMATSGSYTCYRSVQYHNKYYN------KESSAVISVK
658.9 D2       DYLSLKVPP-FVFEGDNIQVSCAGYPGYKA-GDAKLNKENN----FIG-SSGNGSFHIGRVTMATSGSYTCHRAVRYHSLYHN------KESSAVISVK
658.7 D2       DYLSLKVPP-FVFEGDNLQVSCAGYPGYEA-GNAKLYKGNE----FIG-FSGNGSFHIGRVTMATNGSYTCYRLVRHHGLFYH------QESSVYISIK
658.2 D2       GYLFLKVPP-FVFEGDNIQVSCAGYPGYYA-GDAKLNKGDQ----LIG-SSPSGSFHFGRVTMATSGPYTCYRPVWHHSMYYS------QVSSVVISVK
658.8 D2       GYLTLKVPP-FVFEGDNLEVSCAGYPGYYA-GAAKLNKGDQ----FIG-NLSSGNFHIGRVTMATSGPYTCYRPVWHHSMYYS------QVSSVYISVK
658.10 D2      GYLSLKVPP-FVFEGDNLQFSCAGYPGYKA-DTAKLNKGDQ----LIG-FSSSGNFHIGRVTMATSGPYTCYRPVRHDGMYYN------KVSSVVISVK
658.4 D2       DYLSLKVPP-FVFEGDNLQVSCAGYPGYKA-ESAKLYKGDQ----LIG-SSPSGNFHIGRVTMATSGPYTCYRYVWHHSMYHY------KESSVYISVK
658.3 D2       DYLSLKVPP-FVFEGDNLQVSCAGYPGYYG-GNAKLYKGYE----FMA-SSGTGSFQIGRVTMATSGPYTCYRYVWHHSAYHS------KWSSVVISVK
658.12 D2      DYLSLKVPP-FVFEGDNLQVSCAGYPGYKA-DTAKLNKGNQ----LIG-SSPSGNFNFGRVTMATSGSYICYRAVKHHLIYYN------QKSSVYISVK
658.11 D2      GYLALKVPP-YVFEGDNLQVSCAGYPGYYT-DTAKLYKGNQ----LIGGPSSSVNFNFGRVTMATSGSYTCYRAVKHHFIYYN------QESSVYISVK
658.13 D2      GYLTLKVPP-FVFEGDYLEVSCAGYPGYEA-GTARLYKGND---TMIG-SSSTGSLWIGAVAMATSGCYTCYRRVVHHGYSYE------KKSDVYISVK
658.1 D2       DWLALKVPL-YVYEGDDLYVACAGYPGYSA-RNAVLYRGNE----VIASSPSDGKFLVGTANMAMSGSYSCTRQVKHLLIYYS------YSSQAYISVR
658.14 D2      GWLTLKVPG-ILFEGDELSVACAGYPGYSA-RDAVLYRGNE----VIASSPSEAVFLVGTANMATSGSYRCTRQVKDGVIYYS------YSSEEIISVK
XFL2 D2        GYVTLQTTT-TVYEGDSLILRCHNPVFGAS-SGAAFYKDGT----ALKSSSSDSSLSLGTVYKNTSGTYRCSK--SIFNARPV-------TDKAFISVK
CF285478 D2    TWLILQTPP-DVHEGDSLSLRCHSQTPYYT-RNSVFYKNNK----IIESPVSESVLHIGRVDVTASGTYKCKKEFYFDHPYTIGYRT--YTDEKYIFVS
XFL1.8 D2      GWVILQAPS-AVHEGDSLSLRCHSRPGYDT-RNTVFYKINN----IIQSQVSESELHIGRVDVTASGTYKCKKEMYFYYLTGNRYRS--NGAELQVRVQ
XFL1.10 D2     AHVILQVPP-LVFEGDTLSLRCHSYDKFPA-VETTFHKDHK----EIKSLGTASELLVGKVNMETSGLYKCSRRIKSHSSFHN------YSDQVDIVVQ
CF271510 D2    GYLALKVPP-FVFEGDILELSCAAYPGYSA-GEAELHKGDQ----SIG-SSHSGNFNIGRVNMKTSGSYTCYRSVKHHLIYYG------KKSSVYISVK
XFL1.6 D2      GYLVLKVPP-FVFEGDILELSCAAYPGYSA-GDAELHKGDQSIE-SIG-SSHSGNFDIGRVNLKTSGSYTCYRSVKHHLIYYG------QKSSVYISVK
XFL1.4 D2      DYLALKVPP-YVFEGDNVYVFCAGYAGFHA-EKADLKKGNM----FIA-SSPNGSFQIGRVTMATSGSYTCYRTVGYYSPYN-------YVSSADISVK
CF270196 D2    DYLALKVPP-FVFEGDNVYVSCAEYPGYDA-EKADLKKGNM----FIA-SSPNGSFQIGRVTMATSGSYTCNRTVGYYSPYN-------YVSSADISVK
XFL1.1 D2      SWLTLKVPM-FVFEGDELYVSCAGYPGYSA-RDAVLYKDNE----VIGSSPSDADFLVGRANMTTSGLYRCTRQVKDGLIYYS------YASEEQIAVK
XFL1.9 D2      GWLALKVPP-FVFEGDELYVSCAGYPGYSA-RDAVLYKDNE----VIGSSPSDADILVGRANMATSGLYRCTRQVKDGLIYYN------YASEEQIFVR
FCRLB D2       DWLILQVPYAPVFEGEPLVLRCRGWYDKV-VYKLHYYHDGQ----AVRYFHSSANYTVLQARASDSGRYQCSGTMRIPVESAPM-----FSAKVAVTVQ
FCRL6 D2       VWLYLQAWPNPVFEGDALTLRCQGWKNTP-LSQVKFYRDGK----FLHFSKENQTLSMGAATVQSRGQYSCSGQVMYIPQTFTQ-----TSETAMVQVQ
FCRL2 D2       DSLTLVAPSS-VFEGDSIVLKCQGEQNWK-IQKMAYHKDNK----ELSVFKKFSDFLIQSAVLSDSGNYFCSTKGQLFLWDK--------TSNIVKIKVQ
FCRL3 D2       DWLILQALHP-VFEGDNVILRCQGKDNKN-THQKVYYKDGK----QLPNSYNLEKITVN-SVSRDNSKYHCTAYRKFYILDIEV-----TSKPLNIQVQ
FCRL4 D2       DSLILQAPYS-VFEGDTLVLRCHRRRKEK-LTAVKYTWNGN----ILSISNKSWDLLIPQASSNNNGNYRCIGYGDENDVF--------RSNFKIIKIQ
FCRL5 D2       ASLILQAPLS-VFEGDSVVLRCRAKAEVT-LNNTIYK-NDN----VLAFLNKRTDFHIPHACLKDNGAYRCTGYKESCCPV--------SSNTVKIQVQ
FCRLA D2       DWLILQGPAKPVFEGDLLVLRCQAWQDWP-LTQVTFYRDGS----ALGPPGPNREFSITVVQKADSGHYHCSGIFQSPGPGIPE-----TASVVAITVQ
FceRI D2       DWLLLQASAEVVMEGQPLFLRCHGWRNWD-VYKVIYYKDGE----ALKYWYENHNISITNATVEDSGTYYCTGKVWQLDY---------ESEPLNITVI
FcgRI D2       GWLLLQVSSRVFTEGEPLALRCHAWKDKL-VYNVLYYRNGK----AFKFFHWNSNLTILKTNISHNGTYHCSG-MGKHRY---------TSAGISVTVK
FcgRIII D2     GWLLLQAPRWVFKEEDPIHLRCHSWKNTA-LHKVTYLQNGK----DRKYFHHNSDFHIPKATLKDSGSYFCRGLVGSKNV---------SSETVNITIT
FcgRIIb D2     EWLVLQTPHLEFQEGETIVLRCHSWKDKP-LVKVTFFQNGK----SKKFSRSDPNFSIPQANHSHSGDYHCTGNIGYTLF---------SSKPVTITVQ
```

D3

```
658.2 D3.1     ELFSKPEISVNSGSLSQINEGDHMTITCDTELS--PRRATTELQFAFYRNGHNV-QGFNLSNQYGVPSAQLEDSGYYTCEISA-HSGSVKKKSDQKSIQIK
658.3 D3       ELFSKPEISVNSGSLSQINEGDHMTITCDTELS--PHRETTELQFAFYRNGHSV-QGFSLSNQYGVPSAQLQDSGNYTCEISA-HSGSVKKKSDQKSIQIK
658.7 D3       ELFSKPEISGNSGSLGQINEGDHMTITCDTELS--PHRETTELQFAFYRNGHNV-QGFSLSNQYGVPSAQLEDSGNYTCEVQT-PTGSVRKESDPKSIQIK
658.10 D3      ELISKPEISVNSGSLSQINEGDHMTITCDTELS--PHRETTELQFAFYRNGHNV-QGFSLSNQYGVPSAQLEDSGNYTCEVQT-PTGSVRKESDPKSIQIK
946.5 D3.2     ELFTVPPIKVRP---DQVTEGDHMTITCDTELR--PHRETTELQFAFYRNGHNV-QGFSLSNQYGVPSAQLEDSGNYTCEVQT-PTGSVRKRSNMVHIHIQ
946.2 D3.2     ELFLYPQINVSA---DQVTEGDHMTITCDTELR--PHRETTELQFAFYRNGHNV-QGFNSSNQYGVPSAQLEDSGNYTCEVQT-PTGSVRKRSNMAHIHIQ
626.15 D3.3    ELFSYPQINVSS---DQVTEGDHMTITCGTELR--PHRETTELQFAFYRNGHNV-QGFSLSNQYEVPSAQLEDSGNYTCEVQT-PTGSVRKRSSMAHIHIQ
946.4 D3.2     ELVSYLHINVSS---DQVTEGDHMTITCDTELR--PHKATTELQFAFYRNGHNV-QGFSLSNQYGVPSAQLEDSGNYTCEVQT-PTDSKRKRSNMVHIHIQ
626.2 D3.3     ELFSYPQINVSS---DQVTEGDHMTITCDTELR--PHRETTELQFAFYRNGHNV-QGFSLSNQYGVPSAQLEDSGNYTCEVQT-PTGSVRKRSNMVHIHIQ
1107.1 D3.2    ELFSTPQIRVSA---YQVTEGDHMTITCDTKLR--PHRETTELQFAFYRNGHNV-QGFNSSKQYGVPSAQLEDSGNYTCEVQT-PTGSIKKNSNVTHIRIQ
626.15 D37     ELFSTPQIKVRP---DQVTQGDHMTITCDTKLR--PHRETTELQFAFYRNGHNV-QGFSLSNQYGVPSAQLEDSGNYTCEVQT-PTGSVRKRSKVSHIEIQ
946.4 D3.1     ELFSAPQIKVSS---DQMTEGDHMTITCDTELR--PHRATTELQFAFYRNGHNV-QGFSLSNQYGVPSAQLEDSGIYTCEVQT-PTGSVRKRSNVGHIHIQ
626.7 D3       ELFSPPQLKVSS---DQVTEGDHMTITCDTELR--PHRETTELQFAFYRNGHNV-QGFSLSNQYGVPSAQLEDSGNYTCEVQT-PTGSVRKRSSVMTFQIQ
626.8 D3.1     ELFSPPQLKVSS---DQVTEGDHMTITCDTELR--PHRETTELQFAFYRNGHNV-QGFSLSNQYGVPSAQLEDSGKYTCEVQT-PTGSVRKRSREAHIHIQ
946.3 D3.3     ELFPYPQIKVSA---DQVTEGDHMTITCDTKLS--PHRETTELQFAFYRNGHNV-QGFNSSNQYGVPSAQLEDSGNYTCEVQT-PTGSVRKRSNMVHIHIQ
626.2 D3.1     ELFTVPEIKVRP---DQVTEGDHMTITCDTELS--PHRETTELQFAFYRYGHNV-QGFNSSNQYGVPSAQLEDSGNYTCEVQT-PTGSVRKKSREAHIHIQ
626.8 D3.2     ELFTWPKISHLP---DQVTEGDHMTITCDTELS--PHRETTELQFAFYRNGTNV-QGFNSSNQYGVPSAQLEDSGNYTCEVQT-PTGSVRKRSSEAHIHIQ
946.2 D3.1     ELFTFPQIKVSS---DQVTEGDHMTITCDTKLS--PHRETTELQFAFYRNGHNV-QGFNSSNQYGVPSAQLEDSGNYTCEAQT-PTGSVRKRSNVLHIHIQ
626.15 D3.4    ELFNNTQIKVSS---DQVTEGDHMTITCDTKLS--PHRETTELLFAFYRNGHNV-QGFSSSNQYGVPSAQLEDSGNYTCEVQT-PTGSVRKRSNVLYIHIQ
658.16 D3.1    ELFSPPQIKVSA---DQVTEGDHMTITCDTELS--PHRETTELQFAFYRNGHNV-QGFNSSNQYGVLSAQLEDSGNYTCEVQT-PTGSVRKRSNMTHIHIQ
658.17 D3.2    ELFSPPQIKVRP---DQVTEGDHMTITCDTELS--PHRETTELQFAFYRNGHNV-QGFNSSKQYGVPSAQLEDSGNYTCEVQT-PTGSVRKRSNMSHIHIQ
626.15 D3.2    ELFSPPQIKVRP---DQVTEGDHMTITCDTELR--PHRETTELQIAFYRNGHNV-QGFNSSNQYGVPSAQLEDSGKYTCEVQT-PTGSVRKRSSMVHIHIQ
946.1 D3.2     ELFSYPQIQVRP---DQVTEGDHMTITCDTKLS--PHRETTELQFAFYRNGHNA-QGFSLSNQYGVPSAQLEDSGNYTCELQT-PTGSVRKRSSETHIHIQ
946.8 D3.2     ELFSYPTIKVRP---DQVTEGDHMTITCDTKLS--PHRETTELQFAFYRNGHNV-QGFSLSNQYGVPSAQLEDSGNYTCEVQT-PTGSVRKRSREAHIHIQ
946.3 D3.1     VLFSYPQIKVRP---DQVTEGDHMTITCDTEFS--PHRETTELQFAFYRNGHSV-QGFSLSNQYGVPSAQLEDSGKYTCEVQT-PTGSVRKRSREAHIHIQ
658.16 D3.2    ELFSSPQIKVRP---DQVTEGDHMNITCDTELS--PHRETTELQFAFYRNGHNV-QGFSLSNQYGVPSAQLEDSGKYTCEVQT-PTGSVRKRSNMAHIHIQ
946.1 D3.1     ELFSAPQIKVRP---NQVTEGDHMTITCDTELS--PHRETTELQFAFYRNGHNV-QGFSLSNQYEVPSTRLEDSGNYTCEVQT-PTSSVRKRSIEAHIHIQ
1413.1 D3.1    ELFSTPQLKVRP---DQVTEGDHMTITCDTELS--PHRATTELQFAFYRNGHNV-QGFSLSNQYGVPSAQLEHSGKYTCEVQT-PTGSVRKTSSMAHIHIQ
946.6 D3.2     ELFSYPQIKVSS---DQVTEGDHMTITCDTELR--PHRETTELQFSFYRNGHNV-QGFNSSNQYGVPSAQLEDSGNYTCEVQT-PTGSVRKTSRESLIQIK
946.7 D3.2     ELFSYPQIKVSS---DQVTEGDHMTITCDTELR--PHRETTELQFSFYRNGHNV-QGFNSSNQYGVPSAQLEDSGNYTCEVQT-PTGSVRKTSRESLIQIK
626.10 D3      NLFFIPHLKVNP---GDILEGDHMTITCDTKLS--PHRETTELQFAFYRNGHNV-QGFSLSNQYGVSSAQLEDSGNYTCEVQT-PTGSVRKRSNVLHIHIQ
626.11 D3.1    ELFFIPHLKVNP---GDILEGDHMTITCDTKLS--PHRETTELQFAFYRNGHNV-QGFSLSNQYGVPSTQLEDSGNYTCEVQT-PTGSVRKRSSMAHIHIQ
946.5 D3.1     ELFTVPQIKMSP---NQVIEGDHMTITCGTKLS--PHRETTELQFAFYRNGHNV-QGFSLSNQYGVPSVQLEDSGNYTCEVQT-PTGSVRKRSNMAHIHIQ
946.6 D3.1     ELFSTPQIKVSP---DPIMQRDHMTITCDTELS--PRRATTELQFAFYRNGHNV-QGFSLSNQYGVPSTQLEDSGKYTCEVQT-PTGSVRKRSREAHIHIQ
946.7 D3.1     ELFSTPQIKVSP---DPIMQRDHMTITCDTELS--PRRATTELQFAFYRNGHNV-QGFSLSNQYGVPSTQLEDSGKYTCEVQT-PTGSVRKRSREAHIHIQ
946.8 D3.1     ELFSRPQIKVRP---DPVMQRDHMTITCDTKLS--PRRATTELQFAFYRNGHNV-QGFSLSNQYGVPSAQLEDSGKYTCEVQT-PTDSVRKRSREADIHIQ
658.19 D3.3    ELFSSPQIKVRP---DQVIEGDHMTITCDTELS--PHRETTELQFAFYRNGHNV-QGFSLSNQYGVPSAQLEDSGNYTCEVQT-PTGSVRKGSAMAHIHIQ
1131.4 D3.1    ELFSRPKIKVRP---SPLAEGSNMTITCDTALH--PSRITTPLLFAFYINGNTV-REFSLSRIYRVCSVGPGDWGDYSCAVRT-PTGTVLKLSNVT--QIH
1131.5 D3.1    ELFSRPEIKVRP---SPPAEGSNMTITCDTALH--PSRITTPLQFAFYINGNTV-REFSLSKRYRICSVGPGDLGDYSCAVRT-PTGTVLKLSNVTHIQIH
362_1 D3       ELFPRPEIRADK---DGVQGGDVLTLSCHTALN--PARGATQLQFAFYRNGHNV-QGFSSSSNYTIQSARPQDSGNYTCDTAA-PSVSVTKGSPGLSIHIH
362_4 D3       ELFPRPEIRADK---DGVQEGDVLTLSCHTALN--PARGATQLQFAFYRNGHNV-QGFSSSSNYTIQSARPQDSGNYTCDTAA-PSVSVTKGSPGLSIHIH
362_5 D3.2     ELFPRPEIRADK---DGVQEGDVLTLSCHTALN--PARGATQLQFAFYRNGHNV-QGFSSSSNYTIQSARPQDSGNYTCDTAA-PSVSVTKGSPGLSIHIH
362_5 D3.1     ELFPRPQIRADK---DGVQGGDVLTLSCHTALN--PARGATQLQFAFYRNGHNV-QGFSSSSNYTIQSARPQDSGNYTCDTAA-PSVSVTKGSPGLSIHIH
362_2 D3       ELFPRPEIRADK---DGVQGGDVLTLSCHTALN--PARGATQLQFAFYRNGHNV-QGFSSSSNYTIQSARPQDSGNYTCDTAA-PSVSVTKGSPGISIHIH
435.4 D3.2     ELFSTPELNISS---NPIAEDAEMTLNCIT-QR--LIGQNTELQFAFYRNGEKL-QNFSAFNQYHILSTQLADFGVYVCKVQT-LNRSVKKSSQELEIQIQ
435.5 D3.2     ELFSTPELNMSS---NPIAEDSEMTLNCIT-QS--LIGQNTELQFAFYRNGEKL-QNFSAFNKYHILSTQLADSGVYVCKVQT-LNSSVKKSSQVLEIQIQ
435.6 D3.2     ELFSTPELNMSS---YPIAEDAEMTLNCIT-QS--LIGQNTELQFAFYKNGEKV-QNFSAFNKYHILSTQLADSGVYVCKVQT-LNSSVKKSSQELEIQIQ
435.7 D3.3     ELFSTPELNMSS---YPIAEDAEMTLNCIT-QR--LIGQNTELQFAFYRNGEKV-QNFSAFNKYHILSTQLADSGVYVCKVQT-LNSSVKKSSQELEIQIQ
435.6 D3.3     ELFSTPELNMSS---YPIAEDSEMTLNCIT-QK--LIHQNTELQFAFYKNGEKV-QNFSAFNKYHILSTQLADSGVYVCKVQT-LNSSVKKSSQELDIQIQ
658.19 D3.1    ELFSSPQIKVRP---DQVTEGDHMTITCDTKLS--PHRETTELQFAFYRNGHNV-QGFNSSNQYGVPSAQLEDSGKYTCEVQT-PTGSVRKRSYLAYIQIQ
658.19 D3.4    ELFPYPQIKVRP---DQVTEGDHMTITCDTELR--PHRETTELQFAFYRNGHNV-QGFTLSNQYGVPSAQLEDSGNYTCEVQT-PTGSVRKRSSEVDFQLQ
1131.6 D3.1    ELFSRPEIKVRP---SPPAEGSNMTITCDTALH--PSRITTPLLFAFYINGNTV-WEFSLSNQYQVYSAGPGDWGDYSCAVRT-PTGTVLKLSNVTQIQMQ
1107.1 D3.1    ELFSSPQIKVSS---DQVREGDHMTITCDTELS--PHRETTELQFAFYRNGHNV-QGFSLSNQYGVPSAQLEDSGNYTCEVQT-PTGSVRKRSREEHIQIQ
946.3 D3.2     ALVSYPQIKVSP---DQVTEGDHMTITCDTKLS--PHRETTELQFAFYRNEHNV-QGFSLSNQYGVPSAQLEDSGNYTCEVQT-PTGSVRKRSSVEHIQIL
946.8 D3.3     ELVSYLQINVSS---EQVTEGDHMTITCDTELS--PHRETTELQFAFYRNGHNV-QGFNSSNQYGVPSAQLEDSGNYTCEVQT-PTGSVRKRSRESLIQIQ
946.9 D3       ELFPAPQIKVRP---DQVREGDHMTITCDTELR--PHRETTELQFAFYRNGHNV-QGFSLSNQYGVPSAQLEDSGKYTCEVQT-PTGSVRKRSREINIHIQ
946.10 D3.1    ELFPVPQIKVSP---DQVIEGDHMTITCDTKLS--PHRETTELQFAFYRNGHNV-QGFSLSNQYGVPSAQLEDSGNYTCEVQT-PTGSVRKRSREAQIHIQ
626.1 D3       ELFTVREIKATP---DQVREGDHMTITCDTELR--PHRETTELQFAFYRNGHNV-QGFSLSNQYGVPSAQLEDSGKYTCEVQT-PTGSVRKGSNMVHIHIQ
658.13 D3.2    ELFSYPQIKVSA---DQVTE-DHVTITCDTELR--PHRETTELQFAFYRNGHNV-QGFSLSNQYGVPSAQLEDSGNYTCEVQT-PTGSVRKRSREAQIHIQ
946.2 D3       ELFSSPHINVSQ---YYLIEGDHMTITCDTKLS--PHRETTELQFAFYRNGHNV-QGFSLSNQYGVPSVQLEDSGNYTCEVQT-PTGSVRKRSREAHIHIQ
946.15 D3.2    ELFPYPQIQVSP---DQV--GDHMTITCDTQLR--PHRETTELQFAFYRNGHNV-QGFSLSNQYGVPSAQLEDSGNYTCEVQT-PTGSVRKGSNMAHIHIQ
626.4 D3.1     ELFTVPQITLRP---DQVTEGDHMTITCDTELR--PHRETTELQFAFYRNGHNV-QGFGLSNQYGVPSAQLEDSGNYTCEVQT-PTGSVRKRSHIAFMQVQ
626.4 D3.2     ELFTAPHIKVSP---AQVTEGDHMTITCGTELR--PHRETTELQFAFYRNGHNV-QGFSLSNQYGVPSAQLKDSGKYTCEVQT-PTGSVRKMSAMAHIQLE
626.15 D3.1    ELFSPPHINVSP---YDLIEGDHMTITCDTELR--PHRETTELQFAFYRNGHNV-QGFSLSNQYGVPSAQLEDSGNYTCEVQT-PTGSVRKRSSVVTIHIQ
626.15 D3.5    ELFSTPQIKVRP---NPAREGDHMIITCDTELS--PHRETTELQFAFYRNGHNE-QRFSLYNKYGVPSAQLEDSGNYTCEVQT-PTGSVRKRSSMVHIHIQ
658.5 D3       ELFSKPEISVNSGSLSQINEGDHMTITCDTELR--PHRETTELQFAFYRNGHNV-QGFSLSNQYGVPSAQLEDSGYYTCEIRA-QYGSVNKKSDSKIIHII
658.15 D3.2    ELFSAPHINVSQ---YYLIEGDHMTITCDTELS--PHRETTELQFAFYRNGHNV-QGFSLSNQYLVPSAQLEDSGNYTCEVQT-PTGSVRKRSSELDLELQ
435.7 D3.1     ELFSKPEMIANP---RQITEGADGTLTCTTSLS--PARAATELQFAFYKDGRTV-RDFTFYRTFQLSLAQLEHSGDYTCEART-LSHGVRKMSGILKVFIS
1413.1 D3.3    ELFTVPHIKVSS---DQVIRGDHMTITCDTKLS--PHRETTELQFAFYRNGHNV-QGFSLSNQYGVPSAQLEDSGNYTCEVQT-PTGSVRKTSSMAHMQIE
946.11 D3.1    ELFSYPQIKVSF---DPVSG-DHMTITCDTKLS--PHRETTELQFAFYRNGHNV-QGFSLSNQYGVPSAQLEDSGNYTCEVQT-PTGSVRKRSNLAHIHIQ
626.9 D3       ELFTLPQITVSS---DLVIEGDHMTITCDTELR--PHRETTELQFAFYRNGHHV-QGFSLSNQYGVPSAQLEDSGNYTCEVQT-PTGSVRKRSNIVVIQIQ
658.13 D3.3    ELFPYPQIKVSP---NQVREGDHMTITCDTELS--PHRETTELQFAFYRNGHNV-QGFNSSNQYGVPSAQLEDSGNYTCEAQT-PSGSVKKTSDPKSIQIK
658.8 D3       ELFIPPHINVSQ---YYLLEGDHMTITCDTELS--PHRETTELQFAFYRNGHNV-QGFNSSNQYGVPSAQLEDSGNYTCEVQT-PTGSVRKRSDPKNIQVK
1413.1 D3.4    EFFFGTQIKVSH---YPVTEGDNVTITCDTKLN--PRRATTELQFAFYRNGHNV-QGFSLSNQYGVPSAQLEDSGNYTCEVQT-PTDSVRKRSREAHIHIE
946.1 D3.3     VLFTNPQINVSS---DQVTEGDAMNVTCDTELS--SHRETTELQFAFYRNGHNV-QGFRLSNQYGVPSAELEDSGNYTCEVQT-PTGSVRKRSNMADIHIQ
626.16 D3      ELFSSPQIKVRP---DQVTEGDHMTITCDTKLN--PRRATTELQFAFYRNGHNV-QGFSLSNQYGVPSVQLEDSGNYICEVQT-PTGSVRKRSTGVQMQLY
626.3 D3       ELFSSPQIKVRP---DQVTEGDHMTITCDTKLS--PHRETTELQFAFYRNGHNV-QGFRTSNSYTAHHK---DSGNYTCEVQT-TTGSVRKRSKGTYIQMH
658.6 D3       ELFSKPEISVNSGSPSQINEGDPIMFKCDTNLS--PHRETTELQFAFYRNGHNV-QGFSLSNQYGVPSAQLEDSGYYTCEIRA-QYGSVNKKSDPKSIKIK
1413.1 D3.2    ELFSSPQIKVRP---DQVKEGDHMTITCDTKLI--PRRATTELQFAFYRNGHNV-QGFSLSNQYGVPSAKLKDSGNYACKVKN-PSRSVRKRSTMANIQIG
626.14 D3      ELFSVPYVQVNA---DKILQGDHVTITCDTELH--PHRETTELQFAFYRNGHNV-QGFSLSNQYGVPSAQLEDSGNYTCEAQT-PTGSVRKRSNMSYHWIL
658.1 D3.2     ELFSKPVMKVSP---NQVTEGDHMTITCDTELS--PHRETTELQFAFYRNGHNV-QGFSLSNQYGVPSAQLEDSGSYTCEVRM-KNNMVQKRSNEINIQVT
658.1 D3.1     ELFSKPIVNASQ---NHLTEGDHMTITCDTELR--PHRETTELQFAFYRNGHNV-QGFSSSNQYGVSSAQPEDAGKYTCEVRT-KSQTVQKRSNEINIEVT
658.9 D3       ELFSKPEISVNSGSLSQINEGDPIMFKCDTNLS--PRRETTELQFAFYRNGHNV-QGFNSSNQYGVPSAQLEDSGNYTCEVQT-PTGSVRKRSNMAHIHIQ
658.13 D3.1    ELFSKPKISVNSGSLGQINEGDPIMFKCDTNLS--PRRETTELQFAFYRNGHNV-QGFSLSNQYGVPSAQLEDSGNYTCEVQT-PTGSVRKRSNMVHIHIQ
658.11 D3      ELFSTPQIKVRP---DQVIEGDHMTITCDTKLS--PHRETTELQFAFYRNGHNV-QGFSLSNQYLVPSAQLEDSGYYTCEIRA-QSGSVKKKSGGKSIQIT
658.12 D3.2    ELFSSPQIKVST---DQVTEGDRMTITCDTELS--PHRETTELQFAFYRNGHNV-QGFSLSNQYGVSSAQLEDSGYYTCEIRA-QSGIVNKKSDRKSIQIR
658.16 D3.3    ELFSSPQINVSA---NLVTEGDHMTITCDTKLR--PHRETTELQFAFYRNETKV-QEFSTSYEYKILSFALNNSGEYTCEVQA-LNGRVMKRSSMMDFQLQ
1107.1 D3.3    ELFSAPQIKVSP---DQMTEGDHMTITCDTKLS--PHRETTELQFAFYRNETKV-QEFSRFYEYKILSFALNYSGNYTCEVQA-LNGRVRKRSGVFRIYQN
626.2 D3.2     ELFPYPQIQVSP---DQVTEGDHMTMRCDTKLR--LHRETTELQFAFYRNGTMV-QEFSSSYEYRVLSVAPKDSGNYTCEVQT-PTGSVRKGSRVEHIQIL
658.17 D3.3    ELFSTPHIKVSP---DQVTDGDHMTITCDTN-----CKNTTELQFAFYINGRKV-QNFSSSYEYRVPSVAPKDSGRYTCEVKT-PTGSVRKRSSAFYFQLQ
626.5 D3       ELFSTPQIKVRP---AVVVRGDHMTITCDAE----PRRSTTVLQFAFYRNGRNV-QGFSLSNQYGVPSAQLEDSGNYICEVQT-QSGRRRRRSNVITIQIR
```

```
626.6 D3        ELFSPPQIIVRP---AVVVEGDHMTITCDTE----PRRGSTWLQFAFYRNGTNV-QGFSSFNQYGVPSAQLEDSGNYTCEVQT-LLGRRRR-SNGITIQVQ
1131.5 D3.2     EMFSKPKLRVSS---NPITEGSALTLTCGT-TP--STVSTQELQFAFYWDGQEL-QGFTSSNQYRAYSVQLEDSGNYTCEVQH-PAKSINKTSDMSYIQ--
1131.4 D3.2     EMFSKPELRVSS---NPITEGSALTLTCGT-TP--STVSTRELQFTFYWDGQEL-QGFTSSNQYRVYSVQLEDSGNYSCKVQH-PAKSILKTSELFYIQV-
1131.6 D3.2     EMFSEPELTVSS---NPITEGSALTLTCGT-TT--STVSTRELQFAFYWDGQEL-QGFTSSNQYRVYSVQLEDSGNYSCKVQH-PATGISKASEVSYIQA-
1131.5 D3.6     ELFSQPVIRVST---NPVTEGDTLSLTCDTQRG--RYGENTQLVFVFYKGRNEV-QR-NLSSEYRLPSVRVYDSGEYRCAAAT-PDNTVFKWSSPINIQIP
1131.6 D3.4     ELFSQPVIRMNP---NPVTEGDTLSLTCDTQRG--RYGENTQLVFVFYKGKDEV-QR-SLSSEYRLPSVRVQHSGEYRCAAVT-PDNRVYKASLTITIQ--
1131.4 D3.3     EP--L-VIRMNP---YSVTEGDTLSLTCDTQRG--RYGENTQLEFVFYKGRNEV-QR-SLSSEYRLPSVRVYDSGEYRCAAAT-PDNTVYRWSSTITIQIP
1131.6 D3.3     ELFSRPQIRVNP---NPIREGDTLSLTCDTQRG--QYGENTQLVFVFYKGRNEV-QR-SLSSEYRLPSVRVYDSGDYSCVAAT-PDNRVYKESPPITIQ--
1131.5 D3.5     ELFSQPVIRVNP---NPVTEGDALSLTCDTQRG--QYGDNTQLVFVFYKGRNEV-QR-SLSSEFRLPSVQIKHSGEYSCAVVT-PDNRVNKTSSSTTIWIN
1131.5 D3.3     DLFSQPEIRVST---NPVTEGDALSLTCDTQRG--RYGENTQLEFVFYKGKDEV-RR-TLFSEYRLPSVRVQHSGDYSCVAAT-PNNRLYKQSSTKTIKIT
1131.5 D3.4     EL--RPVIRVSP---NTITEEDTMSLTCDTQRG--PNGNNTQLVFVFYKEMNEV-QR-GLSSEYRLPSVRLQHSGVYSCAAAVTPDNTVYRWSSTITIQIQ
1131.4 D3.4     ELFSKPVIKVSP---N--SAGDTLSLTCDTQRG--RYGENTQLEFVFNKGRNEV-QR-SLSSEYRLPSAWAQDSEVFICAAAT-SDNRVYKWSDHITIQRT
435.1 D3        ELFSKPQLQVSS---NETREGANLTITCSTTLT--PARGSTDLRFGFSKDGRST-QELLLSNKLHIRGAQVNQSGYYTCEVEA-VASRIKKMSNTLYIQIQ
626.15 D3.6     ALLTKPQIILSS---KPLAEGDEMTITCDPNLG--NIRNSTELQFAFFKNGLIV-QGFNDSSNYTVPSAQLEESGNYTCEVKK-RSS----VS---QIQVE
1256.2 D3.1     EPFSLPIIRVTP---ESPLEGYGMTISCDTNLS--LVLAPVELHFAFYAYGNMA-RDFNTSDQYTIPVAQQKDSTDYRCEVKT-PDGAVRKSSNETLIEIE
435.2 D3.2     ELFSNPQIKVSP---YPVAEGANMTLICSS-DS--LNGS-APLQFAFLRNGQEV-KEFSSYEKYQIYKTQRDDSGYYSCEVRT-IDGKVKKVSQTLIIHVQ
1256.2 D3.5     ELVSCPNITVIP--SPPLVQGSDVTFTCHATRN--PLEEETELQFAFYRNGENV-QAFGLSDTYHVPSVRLENSGNYSCEVRS-STSDVRKISRGIRVQIK
435.3 D3.1     EPFTKPDITAMP---DPIIEGGKMSLDCKTRVTQQKAASPGKLQFSFYRDGRVA-QNFSKFSNYQVFPVQMKDSGNYSCEVKISSSS-DTKMSKTLTIHIQ
435.3 D3.2     ELFSIPEIRVSP---HPVTPGANLSVTCSTVSK----SDTTKLQFRFYKDRVLV-RNESFFNNYYINSVQLKDSGNYSCDVHI---NGTVKESKEVGIQLQ
435.7 D3.2     APFSYPNIRVSP---DLVIEGDTLSVTCAIDNH--QSSGATKLQFGFYKDKEPV-QKSSLSNRYYVYSAQLADSGNYSCEVKT-LDNRTKKMSQEVNIKVQ
1256.1 D3.3     EFFTAPDIKVSP---NLITEGSDMAITCHVTLT--AQAVPSLMQFALYKDGVLI-HEFGYSNIWKFPPAQRKDSGNYTCGEKI--STGEIRLSRALQVQIL
1131.3 D3.2     EPIPKPEIRVSP---DEITEGSDMSISCHYTPT--SPGESPWLQFALYRDETTL-IEFSSASEWHISPARAEQSGYYTCAVRP--QYGKEQRAQLSIFPYT
1131.4 D3.5     ELIPKPKIRVSP---KVITEGSDMPISC-YCPV--SSQLKPILQFALYKDETTM-IEFSSASEWHISQAQTEQSGYYTCAVRH--WNGREQRSDGVYIHIH
1256.1 D3.1     EFFTAPDIKVSP---DRVTEGSDMSVSCHSIPS--PPSVP--LLYALYKNGQLV-QGFSYSNGWKYTATQKEDSGDYRCAAKT--LTAEKKMSHVLKIQIL
435.2 D3.1     GQFSDPTLSVSP---YQPTEGDEITLVCSMRSE--FNRITSRMMFAFSKNGRVV-QELGSKYIYRIPSVTPKDSGYYTCDVTP--ETQNGTRRASAYVNVL
1131.1 D3       EHLPNPELRVSP---DLITEGSHISLTCHS-----HSMPKGTLAFALYFKGEFQ-HTFSQYNTWHIESVKKEYSGNYTCAVKSSASSKDIKLSNTVNIQIK
1131.3 D3.1     EHLPNPELRVSP---DLITEGSHISLTCHS-----DSLPKDTLEFALYWNGFIL-FTFSKDNTWHTDSVKKEYSGNYTCGVNSSASSKEIKLSNTVNIQIK
1131.2 D3       EHLPNPELRVSP---DLITEGSHISLPCHS-----HSLPKDTLEFALYRDGFIL-HPFAKDNTWHIYSFEKEHSGNYTCAVISSPSPKDIKLSNTVNIHIK
1256.2 D3.2     ELFSYPGIDFSP---YPVTEGEALTVLCDAS----PFAKPTEMLYEFYANGRRV-QKFSPSNTYTVHPVQLKDSGPYLCLAKT-LNGSVEKLEKFGTASFL
1256.2 D3.3     ELFPTPSISVSP---REVLEGSIMTVSCDIKSE--WRGPGN-PRFILYKDSKVV-QSLSAQGEYKVTAAQLHNAGRYECEARY--PSGEAKTSQNNYVTIQ
XFL2 D3         DLFSKPHIQVSS---NETRVGANVTVRCSSTLT--PARRSTELRFVFNKDGRSM-QDIQPSNELHIRGAWVEQSGYYTCEVEA-VASRIKKFSNTLYIQI-
BX842841 D3     DLFSKPHIQVSS---NETRVGANVTVRCSSTLT--PARRSTELRFVFNKDGRSM-QDIQPSNELHIRGAWVEQSGYYTCEVEA-VASRIKKFSNTLYIQIK
XFL1.3 D3.3     ELFSPPQIKVTQ---DQVTEGDHMTITCDTNPR-----NTTELQFAFYINGTVV-QEFSSSHEYRVPSAAPKDSGKYTCEAQT-TTGSVRKRSSELNISYI
XFL1.12 D3.3    ELFSPPQIKVTQ---DQVTEGDHMTITCDTNPR-----NTTELQFAFYINGIVV-QHFSSSHEYRVPSAAPKDSGNYTCEVQS-TTGSVRKRSSGLNISNI
XFL1.1 D3       ELFSKPVMKVNP---NHPTEGDHMTITCDTKLS--PHRETTELQFVFYRNGHNV-QGFSLSNQYGVPSAQLEHSGNYTCEVRT-NNNTVRKTSDEISVQMA
XFL1.5 D3       ELFSKPVMKVNL---NHPTEGDHMTITCDTKLS--PCRATTELQFAFYRNGHNV-QEFSFSNEYDVSSVQLGDSGRYTCEGRT-KKQTVQKRSNEINIQVT
XFL1.9 D3       ELFSKPVMKVNL---NHPTEGDHMTITCDTKLS--PRRATTELQFAFYRNGHNV-QEFSFSNEYDVSSVQLGDSGRYTCEVRT-KKQTVQKRSNEINIQVT
XFL1.7 D3.2     ELVRTPQIKVSP--DDQVTEGDHMTITCDTKLS--PHRETTELQFVFYRNGHNV-QGFSSSNQYGVPSAQLEHSGNYTCEIRD-QSGSVKKESDPKNIRIQ
XFL1.4 D3.3     ELVRTPLDKSQP--RDQVTEGDHMTITCDTKLS--PHRETTELQFAFYRNGHNV-QEFSSSNQYGVPSAQLEDSGNYCCEIRD-QSGSVKKESDPKNIRIQ
XFL1.4 D3.2     EMFSSPQIKVSP--DDQVTEGDHMTITCDTKLS--PHRETTELQFVFYRNGHNV-QGFSSSNQYGVPSAQLEDSGNYTCEIKD-QSGSVKKASDPKSIEIK
XFL1.12 D3.1    ELFPKPVIQVNL---NHSTEGDHMTITCDTKLS--PHRETTELQFVFYRNGHNV-QGFSLSNQYGVPSAQLEDSGNYTCEVQT-PSDSVRKRSSEENIHIQ
XFL1.8 D3.1     ELFTIPQIKVRP---DQVTEGDHMTITCDTKLS--PHRETTELQFVFYRNGHNV-QGFSLSNQYGVPSAQLEDSGNYTCEVQT-PTGRVRKRSNIVHIQIQ
XFL1.3 D3.1     ELFSPPQIKVSS---DQVTEGDHMTITCDTELS--PHRETTELQFVFYRNVHNV-QGFNLSKQYGVPSAQLEDSGKYTCEVQT-TTGSVRKRSNVTHIQIQ
XFL1.10 D3      EMFSLPQIKVSP---DQVTEGDHMTITCDTKLS--PHRETTELQFVFYRNGHNV-QGFSSSNQYGVPSAQLEHSGNYACEVQT-PTGSVRKRSNGQQI---
XFL1.12 D3.2    ELFPYPLIKVSS---DQVTEGDHMTITCDTKLS--PHRETTELQFVFYRNGHNV-QGFSLSKQYGVPSAQMEDSGNYTCDGQT-PTASVRKRSDNINIQVT
CB943873 D3     ELFSTPQITVDL---YSWTEGNQINITCDTKLS--PHRETTELQFVFYRNGHNV-QGFSLSNQYGVPSAQLEDSGNYTCEVQT-PTGSVRKRSNVMTVEIQ
XFL1.6 D3       ELFSKPIISGNS-GQIQINEGDHMTITCDTNLS--PRRATTELQFVFYRNGHNV-QGFSLSNQYGVPSAQLEDSGNYTCEIKD-QSGGVKKESDPKSIQI-
XFL1.8 D3.2     ELFTAPQIIVSQ---GVLVEGDHMTITCDTKLR--PHRETTELQFVFYRNGHNV-QGFSLSNQYGVPSAQLEPSGNYTCEVQT-STGSVRKMSTESLILIQ
XFL1.4 D3.1     ELFSKPVISVNS-GSPNQINEDSILIKCDTKLS--PHRETTELQFAFYRNGHNV-QGFSSSNQYGVPSAQLEHSGNYTCEIKD-QSGSVKKASDQKSIEIK
XFL1.3 D3.2     ELFSTPQIKVTP---DPVTEGDHMTITCDTN----P-RNTTELQFAFYINGTQV-QGLDSSSYYTVSLAQMNNSGKYNCVVQT-ASGSVKKNSSETHIHIQ
XFL3 D3         ESLPKPQLRVSP---DVITEGTNVTLTCHSDLL---HKDTT-LEFGLYWNGFIS-YIFSKDNTWHIYSFKKEFCGNYTCGVNSSTSPKDIIMSDVVNIQI-
BU903031 D3     ELFSIPEIKLIS---HPVIPGANLSITCSTVSK----SVATQPRFRFYKDQVLV-QNESLSNNYYMNSVQLMDSGNYSCDARI---YGTVKTSSQVSVQVQ
FcRI D3         ELFPAPVLNASVT–SPLLEGNLVTLSCETKLL-LQ--RPGLQLYFSFYMG-SKTLRGRNTSSEYQILTARREDSGLYWCEAAT-EDGNVLKRSPELELQVL
FCRLB D3        ELFRAPVLRVMGP--REARGAGGVVLRCDTRLH--PQKRDTPLQFAFYKY-SRAVRRFDWGAEYTVPEPEVEELESYWCEAAT-ATRSVRKRSPWLQLPGP
FCRL6 D3        ELFPPPVLSAIPS--PEPREGSLVTLRCQTKLHPL--RSALRLLFSFHKD-GHTLQDRGPHPELCIPGAKEGDSGLYWCEVAP-EGGQVQKQSPQLEVRVQ
FCRLA D3        ELFPAPILRAVPS--AEPQAGSPMTLSCQTKLPLQ--RSAARLLFSFYKD-GRIVQSRGLSSEFQIPTASEDHSGSYWCEAAT-EDNQVWKQSPQLEIRVQ
FCRL2 D3        ELFQRPVLTASSF---QPIEGGPVSLKCETRLSPQ--RLDVQLQFCFFRENQVLGSGWSSSPELQISAVWSEDTGSYWCKAET-VTHRIRKQSLQSQIHVQ
FCRL4 D3        ELFPHPELKATDS---QPTEGNSVNLSCETQLPPE--RSDTPLHFNFFRDGEVILSDWSTYPELQLPTVWRENSGSYWCGAET-VRGNIHKHSPSLQIHVQ
FCRL3 D3        ELFLHPVLRASSS---TPIEGSPMTLTCETQLSPQ--RPDVQLQFSLFRDSQTLGLGWSRSPRLQIPAMWTEDSGSYWCEVET-VTHSIKKRSLRSQIRVQ
```

D4

```
658.11 D4   AQNLAGVRVRLEPAGGQVIAGEKLEILCSVEKGMG--LLRYSWCKQPTLSCVTKEAAASEQ-RFVVESVSEDYGGEYRCIVTRTATGESMRSANISISVQ
658.3 D4    AQNLAGVRVRLEPAGGQVIAGEKLEILCSVEKALG--LLRYSWCKQPTRICDTKEAAASEQ-RFVVESVSEDYGGEYQCIVTKADTGESIRSANISISVP
658.12 D4   AKKLAGVRVRLEPAGGQVIAGEKLEILCSVEKGMG--LLRYSWCKQPTLICNTKEAAAPEQ-RFVAESVSEDYGGEYQCIITRTATGESIRSANISISVQ
658.2 D4    AQTLAGVRVRLEPAGGQVIAGEKLEILCSVEKAMG--LLRYSWCKQPTRICDTKEAAASEQ-RFVVESVSEDYGGEYQCTVTRAATGESMRSANISISVR
658.1 D4    AQQASEVTVRLEPPQGQVYAGEKLEVFCSVDKWVG--SLIFSWCKHKRTACEEKRASSQEQ-HFVVEAVPEGYGGEYQCIVTTEDTKVSIRSTNTWISVS
XFL1.6 D4   AQKLSDVSVRLESAGGQVIAGERLEIICSVEKGME--LLRYSWCKQSSLSCDTKESSASEQ-RFVVERVPEDYGGQYQCTVARVATGNSMRSRKFSISVR
XFL1.5 D4   AQQASKVTVRLEPPQGQIYAGEKLEVFCSVDKVAD--SLTFSWCKHNELHCEGKTARSKEQ-HFVVEAVPEDYSEEYQCIVTRERTQDSIRSTKIKISVS
XFL1.7 D4   AQKLSGVSVRLEPAGGQVLAGEKLEILCSVEKGMG--LLSYSWCKQSSLSCDTKEAAVLEQ-RFVVENVSEDYGGEYQCIVTRTATGDSISSANISISIS
XFL1.11 D4  AQKLSGVRVSLEPTGEQVIAGEKLEILCSVEKGMG--LLSYSWCKQYNLSCDTKEATALEQ-RFVVESVSEDYGGEYQCIVTRAATQDSIQSANISISVQ
XFL1.9 D4   AQQASEVTVRLEPPQGQIYAGEKLEVFCSVDKVAD--SMTFSWCKHNELHCEGKTARSKEQ-HFVVEAVPEDYSEEYQCIVTRERTQDSIRSTKIKIYVS
FCRL2 D4    RIPISNVSLEIRAPGGQVTEGQKLILLCSVAGGTG--NVTFSWYREATGTSMGKKTQRSLSAELEIPAVKESDAGKYYCRADNGHV--PIQSKVVNIPVR
FCRL3 D4    RVPVSNVNLEIRPTGGQLIEGENMVLICSVAQGSG--TVTFSWHKEGRVRSLGRKTQRSLLAELHVLTVKESDAGRYYCAADNVHS--PILSTWIRVTVR
FCRL1 D4    RVPVADVSLETQPPGGQVMEGDRLVLICSVAMGTG--DITFLWYKGAVGLNLQSKTQRSLTAEYEIPSVRESDAEQYYCVAENGYG--PSPSGLVSITVR
FCRL4 D4    RIPVSGVLLETQPSGGQAVEGEMLVLVCSVAEGTG--DTTFSWHREDMQESLGRKTQRSLRAELELPAIRQSHAGGYYCTADNSYG--PV-QSMVLNVTV
```

D5

```
658.5 D5      VKV---ELKLSPT--KVAVGDSVDLLCESKSGSFPVDYQFYHRNDSIGKGKAKKKEEAQYRVTITSISMAGPYYCTLQNEFSSKMQLSVGVILSVM
658.11 D5     VKVGKPELKLSPT--KVAVGDSVDLLCESKSGSFPVDYQFYHRNDSIGKGKAKKKEEAQYRVTITSTSMAGPYYCALQNEFSSKMQLSDGVNLSVM
658.2 D5      VKVGKPELKLSPT--KVAVGDSVDLLCESKSGSFPVGYQFYHRNDSIGKGKAEKKEEAQYRVTITSISLAGPYYCALQNEFSSKMQLSEGVILSVM
658.1 D5      VKVRIPLLRVSPK--EVAVGDTVNLLCESKSRSFPKEYQFYHMEDIIGSIKGPQKAATQLNVTITSLTMAGPYLCAVRNDVSSTLRYSEGVTLS--
XFL1.6 D5     VRVKLGKPELKLSPGKVAVGDSVDLFCESKSGSFPVDYQFYHRNDSVGKGKAEKKENAKYRVTVTSITMTGPYSCALQNEVSSKMQLSEGLVLSVM
XFL1.5 D5     VSVRLPLLTVSLK--EVAVGDIVVLLCESKSRFFPKEYQFYHMEDFLGSIKGDQKAAAQFNVTITSLTMAGPFICVVQNNVFSPK--SEGVTLFVM
FCRL6 D5      APVSRPVLTLHHGPADPAVGDMVQLLCEAQRGSPPILYSFYLDEKIVGNHSAPCGGTTSLLFPVKSEQDAGNYSCEAENSVS--RERSEPKKLSLK
FCRL1 D5      IPVSRPILMLRAPRAQAAVEDVLELHCEALRGSPPILYWFYHEDITLGSRSAPSGGGASFNLSLTEEH-SGNYSCEANNGLG--AQRSEAVTLNFT
FCRL2 D5      IPVSHPVLTFRAPRAHTVVGDLLELHCESLRGSPPILYRFYHEDVTLGNSSAPSGGGASFNLSLTAEH-SGNYSCDADNGLG--AQHSHGVSLRVT
FCRL3 D5.1    VPVSRPVLTLRAPGAQAVVGDLLELHCESLRGSFPILYWFYHEDDTLGNISAHSGGGASFNLSLTTEH-SGNYSCEADNGLG--AQHSKVVTLNVT
FCRL5 D5.1    IPASHPVLTLSPEKALNFEGTKVTLHCETQEDSLRTLYRFYHEGVPLRHKSVRCERGASISFSLTTEN-SGNYYCTADNGLG--AKPSKAVSLSVT
FCRL5 D5.6    VPVSRPVLTLRAPGTHAAVGDLLELHCEALRGSPLILYRFFHEDVTLGNRSSPSGG-ASLNLSLTAEH-SGNYSCEADNGLG--AQRSETVTLYIT
```

D6

```
658.13 D6 EPVADARITPGEDELAVIVEDNLCLTCSVAKGTNPLFLWIYNNETIGHESVL--YQVR-ESGKVMCIASAQLQHAGTYWCQVSNKLWDNRIFTVTSNIVTISIS
658.1 D6  EPVANVTISPGMDVLEVRAENSLCLTCSVAKGTSPSLLWVYNNENVDQVPISGSYQVEFESEKVLCINSVQWYHTGVYQCQASNQLSPNRIFTAESNIVTITIT
658.12 D6 EPVADARITPEEDELAVKVEDNLCLTCSVAKGTN-LFLWIHNNKTIGHESVL--YQVR-ESGKVLCIESAQLQHAGTYWCEVRNQLSSNRIFTVTSNIVTISIS
658.5 D6  EPVADARITPGEDELAVIVEDNLCLTCSVAKGTNPLFLWIYN-ETIEHESVL--YQVR-ESGKVLCIESAQLQHAGTYWCQVRNQLSSNRIFSVTSNNVTISIS
658.7 D6  VPVADARITPGEDELAVKVEDNLCLTCSVAKGTNPLFLWIYNNETIGHESVL--YQVR-ESGKVMCIESAQLQHAGSYWCRVSNQLWDNRIFTVTSNNVTISIS
658.2 D6  EPVADARITPGEDELAVILEDNLCLTCSVAKGTNPRFLWFYNNET-EHESVL--YQVR-ESGKVLCIESAQHQHSRTYWCQVSNQLSSNRIFTVTSNNVTISIS
658.11 D6 EPVADARITPGEDELAVIVEDNLCLTCSVAKGTNPLFLWFYNNETIEHESVL--YQVR-ESGKVLCIESAQLQHAGTYWCQVRNQLSSNRIFSVTSNNATISIS
```
